# Lentivirus‐mediated gene therapy for Fabry disease: 5‐year End‐of‐Study results from the Canadian FACTs trial

**DOI:** 10.1002/ctm2.70073

**Published:** 2025-01-10

**Authors:** Aneal Khan, Dwayne L. Barber, William M. McKillop, C. Anthony Rupar, Christiane Auray‐Blais, Graeme Fraser, Daniel H. Fowler, Alexandra Berger, Ronan Foley, Armand Keating, Michael L. West, Jeffrey A. Medin

**Affiliations:** ^1^ M.A.G.I.C. (Metabolics and Genetics in Canada) Clinic Calgary Alberta Canada; ^2^ Department of Laboratory Medicine and Pathobiology University of Toronto Toronto Ontario Canada; ^3^ Department of Pediatrics Medical College of Wisconsin Milwaukee Wisconsin USA; ^4^ Department of Pathology and Laboratory Medicine Western University London Ontario Canada; ^5^ Department of Pediatrics, Division of Medical Genetics CIUSS de l'Estrie‐CHUS Hospital Fleurimont University de Sherbrooke Sherbrooke Quebec Canada; ^6^ Department of Oncology McMaster University and Juravinski Hospital and Cancer Centre Hamilton Ontario Canada; ^7^ Rapa Therapeutics Rockville Maryland USA; ^8^ Princess Margaret Cancer Centre University Health Network Toronto Ontario Canada; ^9^ Department of Pathology and Molecular Medicine McMaster University and Juravinski Hospital and Cancer Centre Hamilton Ontario Canada; ^10^ Division of Nephrology, Department of Medicine Dalhousie University Halifax Nova Scotia Canada; ^11^ Department of Biochemistry Medical College of Wisconsin Milwaukee Wisconsin USA

**Keywords:** clinical trial results, haematopoietic stem cells, lysosomal storage disorders, melphalan conditioning, progenitor cells

## Abstract

**Background:**

Fabry disease is an X‐linked lysosomal storage disorder due to a deficiency of α‐galactosidase A (α‐gal A) activity. Our goal was to correct the enzyme deficiency in Fabry patients by transferring the cDNA for α‐gal A into their CD34+ hematopoietic stem/progenitor cells (HSPCs). Overexpression of α‐gal A leads to secretion of the hydrolase; which can be taken up and used by uncorrected bystander cells. Gene‐augmented HSPCs can circulate and thus provide sustained systemic correction. Interim results from this ‘first‐in‐the‐world’ Canadian FACTs (Fabry Disease Clinical Research and Therapeutics) trial were published in 2021. Herein we report 5‐year ‘End‐of‐Study’ results.

**Methods:**

Five males with classical Fabry disease were treated. Their HSPCs were mobilized, enriched, and transduced with a recombinant lentivirus engineering expression of α‐gal A. Autologous transduced cells were infused after conditioning with a nonmyeloablative, reduced dose, melphalan regimen. Safety monitoring was performed. α‐Gal A activity was measured in plasma and peripheral blood (PB) leucocytes. Globotriaosylceramide (Gb3) and lyso‐Gb3 levels in urine and plasma were assessed by mass spectrometry. qPCR assays measured vector copy number in PB leucocytes. Antibody titers were measured by ELISA. Body weight, blood pressure, urinary protein levels, eGFR, troponin levels, and LVMI were tracked.

**Results:**

Four out of 5 patients went home the same day as their infusions; one was kept overnight for observation. Circulating α‐gal A activity was observed at Day 6–8 in each patient following infusion and has remained durable for 5+ years. LV marking of peripheral blood cells has remained durable and polyclonal. All 5 patients were eligible to come off biweekly enzyme therapy; 3 patients did so. Plasma lyso‐Gb3 was significantly lower in 4 of 5 patients. There was no sustained elevation of anti‐α‐gal A antibodies. Patient weight was stable in 4 of the 5 patients. All blood pressures were in the normal range. Kidney symptoms were stabilized in all patients.

**Conclusions:**

This treatment was well tolerated as only two SAEs occurred (during the treatment phase) and only two AEs were reported since 2021. We demonstrate that this therapeutic approach has merit, is durable, and should be explored in a larger clinical trial.

**Highlights:**

- This was the first gene therapy clinical trial to be completed for Fabry disease.
- There were no adverse events of any grade attributable to the cellular gene therapy intervention or host conditioning throughout the follow‐up interval of 5 years.
- After reduced‐intensity melphalan treatment, all patients engrafted their autologous modified α‐gal A expressing cells.
- All patients synthesized and secreted α‐gal A throughout the course of the study.
- Expression of α‐gal A resulted in a decrease in plasma lyso‐Gb3 in four of five patients and stabilization of kidney symptoms in all patients.

## Graphical abstract

Durable 5‐year End‐of‐Study results from the ‘first‐in‐the‐world’ gene therapy trial for Fabry disease.

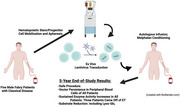

## 1 INTRODUCTION

Fabry disease (OMIM 301500) is an X‐linked lysosomal storage disorder (LSD) originally found to be due to impaired hydrolysis of the terminal galactose of the sphingolipid ceramide trihexoside (globotriaosylceramide, Gb_3_) by the enzyme alpha‐galactosidase A (α‐gal A), encoded by the *GLA* gene.^1^, ^2^ The realisation that it was a lack of the enzyme activity resulting in accumulated sphingolipids with terminal galactose residues has been the mainstay of understanding this disease. Principal treatments have involved replacement of this enzyme activity using various products or enhancement of misfolded lysosomal activity using chaperones.^3^, ^4^, ^5^, ^6^ Therapeutic goals focus on reducing the clinical burden of the disorder from cardiac dysfunction and renal insufficiency, along with reducing the higher risk of stroke and lessening chronic pain.^7^ Since the start of enzyme replacement therapy (ET), this method of treatment has been the most commonly used worldwide. ET involves biweekly enzyme infusions, with products that have a half‐life of less than 2 h.^8^, ^9^ A pegylated form of the enzyme has been approved in the United States that has demonstrated a half‐life between 53 and 121 h,^10^ which still requires biweekly intravenous infusions. An oral chaperone (migalastat) has also been approved but only certain *GLA* mutations are amenable.^4^ Migalastat was not approved for clinical use at the time of initiation of this study. The use of ET means a biweekly intravenous infusion for life, requiring infusions at a hospital, infusion centre or at home, repeated vascular access, the potential use of central lines and the complications that accompany them. Reimbursement issues are problematic for intravenous or oral drugs. Patients may not be able to move to a geographical region where coverage is provided thereby limiting lifestyle choices and career goals. As an alternative to the burden and cost of lifelong use of medication, this prompted a need for a ‘one and done’ approach. There are two broad approaches to gene therapy in this context: lentiviral or adeno‐associated virus (AAV) modalities. An ex vivo lentiviral approach, which transduces multi‐potent haematopoietic stem cells, such as CD34^+^ lymphocytes, that populate the bone marrow, can provide systemic production of α‐gal A. Direct AAV injection has a higher tropism for the liver, but this approach cannot be used in patients who have a cross‐reactive antibody to the AAV vector serotype being used. Since the AAV transduction is primarily performed in non‐dividing cells, the effective dose of the vector can also reduce over time. In Fabry disease, α‐gal A can be taken up by uninfected bystander cells, similar to exogenous intravenous ET, to provide cross‐correction in those cells.

The FACTs (Fabry Disease Clinical Research and Therapeutics) study involved recombinant lentivirus (LV)‐mediated transfer of a codon‐optimised cDNA for human α‐gal A into haematopoietic stem/progenitor cells (HSPCs) from Fabry patients followed by autologous re‐infusions of the cell products (clinicaltrials.gov, #NCT02800070). The study was a non‐randomised, open‐label, multi‐centre clinical trial to evaluate the safety and efficacy of LV‐transduced HSPCs for 5 years in males with classical Fabry disease who were already on ET. The primary endpoints (safety) were determined using criteria from the National Cancer Institute of Canada (NCIC) Common Terminology Criteria for Adverse Events (CTCAE). Safety monitoring assessments were performed for cardiac and renal function as well as for stroke, which are the three principal clinical outcome‐related organ systems in Fabry disease. Secondary analyses included: (a) changes in α‐gal A enzyme activity within the plasma, lymphocytes and bone marrow, (b) changes in Gb_3_ levels in plasma and urine, (c) changes in lyso‐Gb_3_ and related analogue levels in plasma and urine, (d) persistence of LV‐transduced cells as measured by quantitative polymerase chain reaction (qPCR) and (e) determination of anti‐α‐gal A antibody titres.

Interim results and safety data were published in 2021.^11^ In addition to the interim publication on safety and clinical outcomes, a recent paper showed sustained reductions in Gb_3_ and globotriaosylsphingosine (lyso‐Gb_3_) levels in our treated Fabry patients.^12^ Longitudinal evaluations of engraftment in our Fabry disease patients receiving LV‐mediated gene therapy were also performed; there was evidence for persistent polyclonal haematopoiesis and no evidence of clonal dominance in any patient.^13^ Examining clonal expansion is relevant because lentiviral constructs integrate into host DNA and have the potential to dysregulate proto‐oncogenes, which can manifest with clonal expansion and haematological malignancies.^14^ Herein, we present the 5‐year End‐of‐Study results from that FACTs trial, with ongoing safety monitoring outcomes, adverse clinical event assessments, and renal and cardiac function analyses.

## 2 RESULTS

### 2.1 α‐Gal A activity and vector copy number

Our study design was described in an earlier interim report.^11^ This open‐label clinical trial was divided into four phases: Phase 1 (screening), Phase 2 (mobilisation), Phase 3 (treatment), and Phase 4 (post‐treatment follow‐up). Seven males with classic Fabry disease were screened for participation in the trial; five met the inclusion and exclusion criteria in order to proceed. All patients signed informed consent. Our earlier report characterised the initial results for all five patients up to 18 months post‐infusion.^11^ Patients treated with autologous CD34+ cells engineered to express α‐gal A were shown to synthesise the missing enzyme in their leukocytes resulting in secretion of the deficient enzyme into their plasma. This resulted in continuous enzyme production and a decrease in glycosphingolipid accumulation, most notably plasma lyso‐Gb_3_. Antibody titres to α‐gal A were shown to decrease after gene therapy. This End‐of‐Study report analyses subjects for 5 years post‐treatment.

**FIGURE 1:**
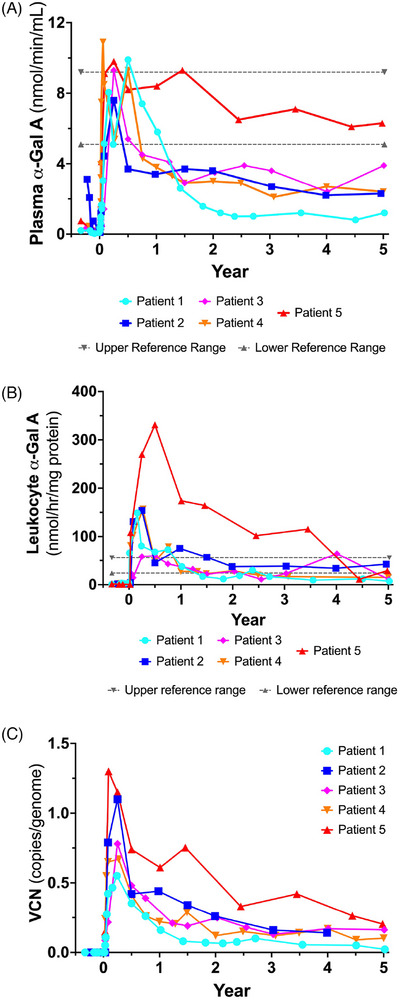
α‐Galactosidase A (α‐gal A) activity and vector copy number (VCN). (A) Plasma α‐gal A activity is illustrated for each patient. The reference ranges (dotted line) were defined by Dr. Rupar's laboratory from 150 normal specimens referred for diagnostic testing. The lower reference range is 5.1 nmol/min/mL and the upper reference range is 9.2 nmol/min/mL. (B) Leukocyte α‐gal A‐specific activity is shown from the identical time points as illustrated in (A) above. The reference ranges (dotted line) were defined by Dr. Rupar's laboratory from 150 normal specimens referred for diagnostic testing. The lower reference range is 24 nmol/h/mg protein and the upper reference range is 56 nmol/h/mg protein. (C) VCN is presented for each patient. There was no data point for year 5 of Patient 2 due to a technical problem with this sample.

We have elected to focus on results from years 1 to 5 in this report, as earlier time points were previously described.^11^ Plasma α‐gal A activity was shown to be significantly increased (Supporting Information Figure 1) in all five patients from 1 to 5 years after stem cell transplantation (Figure 1A). Plasma levels were elevated at 1 year, then reached asymptotes throughout the course of the study. All patients were below the reference range for plasma α‐gal A activity, with the exception of Patient 5. Leukocyte α‐gal A‐specific activity showed a similar response to plasma α‐gal A activity (Figure 1B). Stable hydrolase specific activity was significantly increased post‐therapy from years 1 to 5 of the study (Supporting Information Figure 2). In this case, all patients were within or slightly below the reference range, with the exception of Patient 5 whose leukocyte α‐gal A‐specific activity was supranormal.

Vector copy number (VCN) was also analysed in white blood cells obtained from all treated patients. The methods for VCN determination have been described previously.^15^ VCN was significantly increased in all patients (Supporting Information Figure 3). VCN decreased more rapidly at earlier time points as short‐term HSPCs were extinguished, but the rate of decline was more moderate from years 1 to 5 of this study (Figure 1C). VCN mirrors leukocyte α‐gal A activity as we previously reported.^11^

### 2.2 Clinical findings

Patient weight was shown to be stable in Patients 1–4 after the treatment phase (Supporting Information Figure 4). Patient 5 gained weight throughout the trial unrelated to study procedures or product. He was initially 142 kg during Phase 1, but reached 170 kg by year 5 of the study. Blood pressure data were analysed in Phases 1 and 4 of the trial. Systolic blood pressure dropped in Patients 1 and 4, whereas increases in systolic blood pressure were documented in Patients 2, 3 and 5 (Supporting Information Figure 5). Diastolic blood pressure decreased in Patients 1, 2 and 5, while higher diastolic blood pressures were observed in Patients 3 and 4 (Supporting Information Figure 5). All blood pressure measurements were in the normal range, but the increase in slope in Patient 5 is being actively monitored and may be related to factors associated with weight gain.

### 2.3 Renal outcomes

**FIGURE 2:**
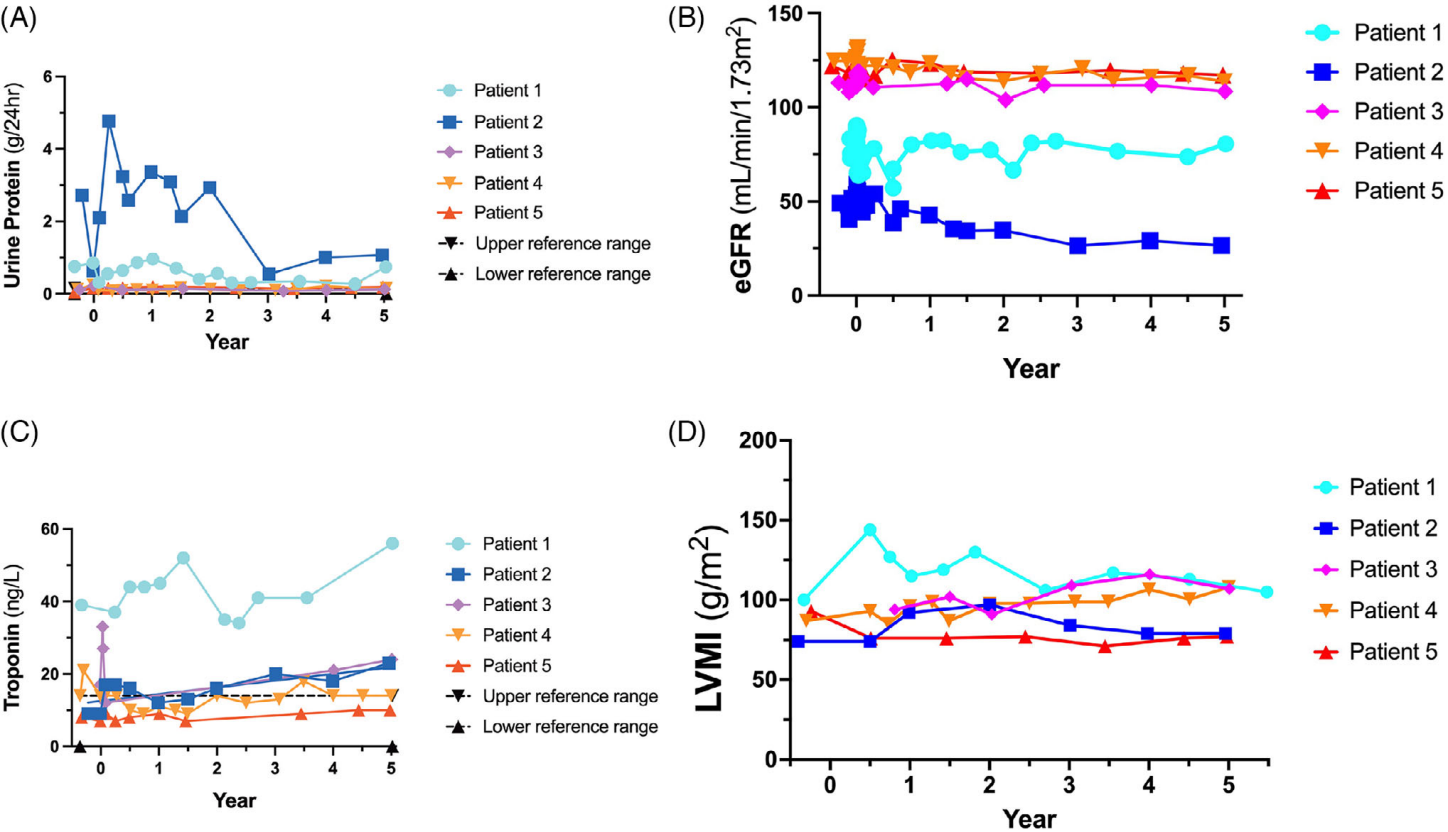
Clinical outcome parameters. (A) Urinary protein secretion is illustrated throughout the trial for all patients. Lower (0 g protein/24 h) and upper (.15 g protein/24 h) reference ranges are represented as dashed grey lines. (B) Estimated glomerular filtration rate (eGFR) is shown for all patients. eGFR was calculated using the chronic kidney disease‐epidemiology collaboration formula. (C) Troponin levels were followed for all patients during the trial. Lower (0 ng/L) and upper (14.0 ng/L) reference ranges are depicted as dotted grey lines. (D) Left ventricular mass index (LVMI) was monitored during the trial for all patients. eGFR was calculated using the chronic kidney disease‐epidemiology collaboration formula. Values from magnetic resonance imaging (MRI) are shown for LVMI in (D). Patient 1 was unable to attend a year 5 End‐of‐Study appointment until year 5.5 due to COVID‐related travel restrictions.

Urinary protein levels, collected over 24 h, were determined for all patients (Figure 2A). Patient 2 had elevated initial measurements far exceeding the reference range (0–.15 g/24 h). However, these levels were observed to decrease throughout the trial to year 3 at which point stabilisation was observed, almost approaching the level at the time of transplantation. Patient 2 had Stage 3 chronic kidney disease at the time of enrolment. Urinary protein decreased for Patient 1 during years 2–4.5, but raised to pre‐transplant levels by year 5. Patients 3–5 all had low levels of protein excretion near the upper reference range. There was a biphasic slope for urinary protein for Patient 2 in this study (Supporting Information Figure 6). A slope of −.37 was observed until year 3, at which point there was stabilisation and a slope of .27 was observed for the final 2 years of Phase 4. Because of the small sample size, there is no specific reason we could attribute to stabilisation of the proteinuria 3 years post‐transplant in Patient 2. The slope calculations ranged from −.06 (Patient 1) to −.01 (Patient 3), .003 (Patient 4) and  .006 (Patient 5). All patients maintained use of other medications during this period. Patients 1 and 2 were prescribed losartan, but due to the voluntary recall of that drug during the FACTs study, they were switched to an equivalent dose of irbesartan. By comparison, the annualised mean change in estimated glomerular filtration rate (eGFR) in patients with Fabry disease treated with migalastat was −.7 mL/min/1.73 m^2^/year^16^ and −.97 mL/min/1.73 m^2^/year in healthy male controls who initially had normal kidney function.^17^ The therapeutic target is up to ‐3 mL/min/1.73 m^2^/year^7^ and can be slightly less than this in patients on ET.^18^

**FIGURE 3:**
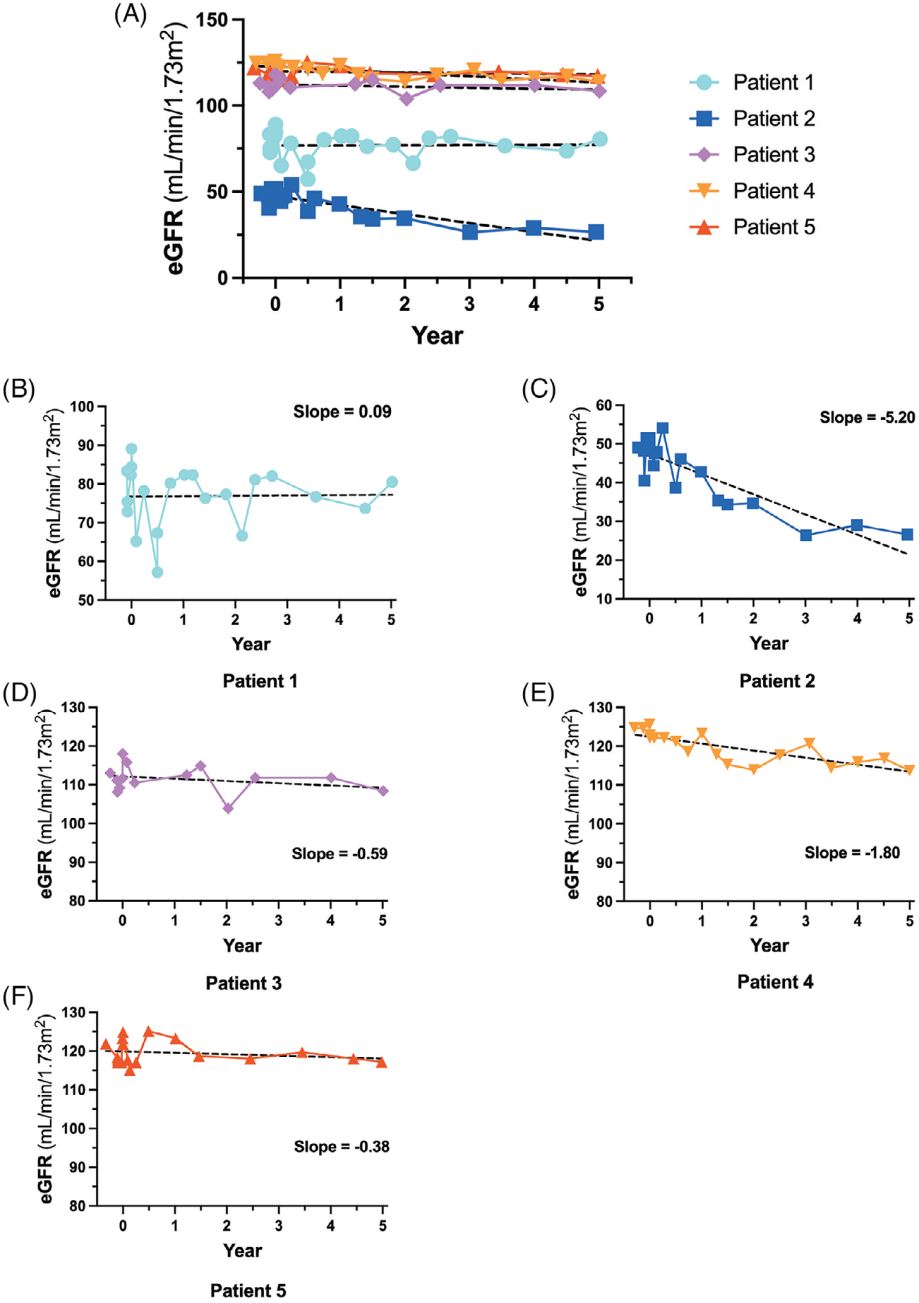
Estimated glomerular filtration rate (eGFR) slope. eGFR data including all data points with the exception of Phase 3 (treatment phase from day 0 to 33) are shown. The eGFR was divided by the total study period in years in order to calculate eGFR/year. Linear regression was performed to calculate slopes.

As proteinuria is associated with kidney decline, we calculated eGFR for all patients throughout the study (Figure 2B). Treatment phase data were omitted for clarity as melphalan treatment resulted in a transient increase in eGFR activity (data not shown). Patient 2 displayed Fabry‐associated kidney disease and had an eGFR level that decreased throughout the study until year 3 when stabilisation was observed. Patient 1 had a more intermediate eGFR reading, consistent with Stage 2 kidney disease in this subject. Finally, Patients 3–5 had stable eGFR measurements near 90 mL/min/1.73 m^2^. eGFR slope is a documented marker of kidney function over time.^19^ Patient 2 demonstrated a biphasic response with a slope of −7.48 until year 3 and then a slope = .11 from years 3 to 5 of this study, similar to the 24 h urinary protein results illustrated in the previous figure (Figure 3C). Patients 3–5 had eGFR slopes that were decreasing (Patient 3 = −.59, Patient 4 = −1.80 and Patient 5 = −.38). In contrast, the eGFR slope for Patient 1 was closer to 0 (.09; Figure 3B).

### 2.4 Cardiac outcomes

Troponin is a sensitive biomarker that monitors myocardial inflammation.^20^ Patient 1 had elevated troponin levels (Figure 2C) that increased throughout this study with a slope of 1.55 (Supporting Information Figure 7). Patient 2 also displayed a positive slope (1.87) with a troponin concentration of 23 ng/L at year 5. Patients 4 and 5 were in the reference range (0–14 ng/L). Patient 4 had a slope of .07 whereas the troponin slope for Patient 5 was .42.

Patients 1, 2 and 3 had a transient increase in left ventricular mass index (LVMI) with an eventual reduction in LVMI by 5 years (Figure 2D). This occurred despite Patients 1 and 2 remaining on ET during the rise in LVMI post‐gene therapy. At 5 years, Patient 1 had the same LVMI compared to baseline despite being off ET for 3.5 years. There was no change in T1 mapping (Patients 1, 2, 3 and 5) or the degree of late gadolinium enhancement (LGE), indicating fibrosis in any patient (data not shown). Patient 4 did not have T1 mapping performed since this methodology was not available at all regional stem cell centres. Patient 1 developed intermittent atrial fibrillation with an intra‐atrial thrombus but was treated with anticoagulation and remains in the same general health with no other changes on cardiac MRI at baseline or at 5 years (data not shown).

### 2.5 Globotriaosylceramide (Gb_3_)

**FIGURE 4:**
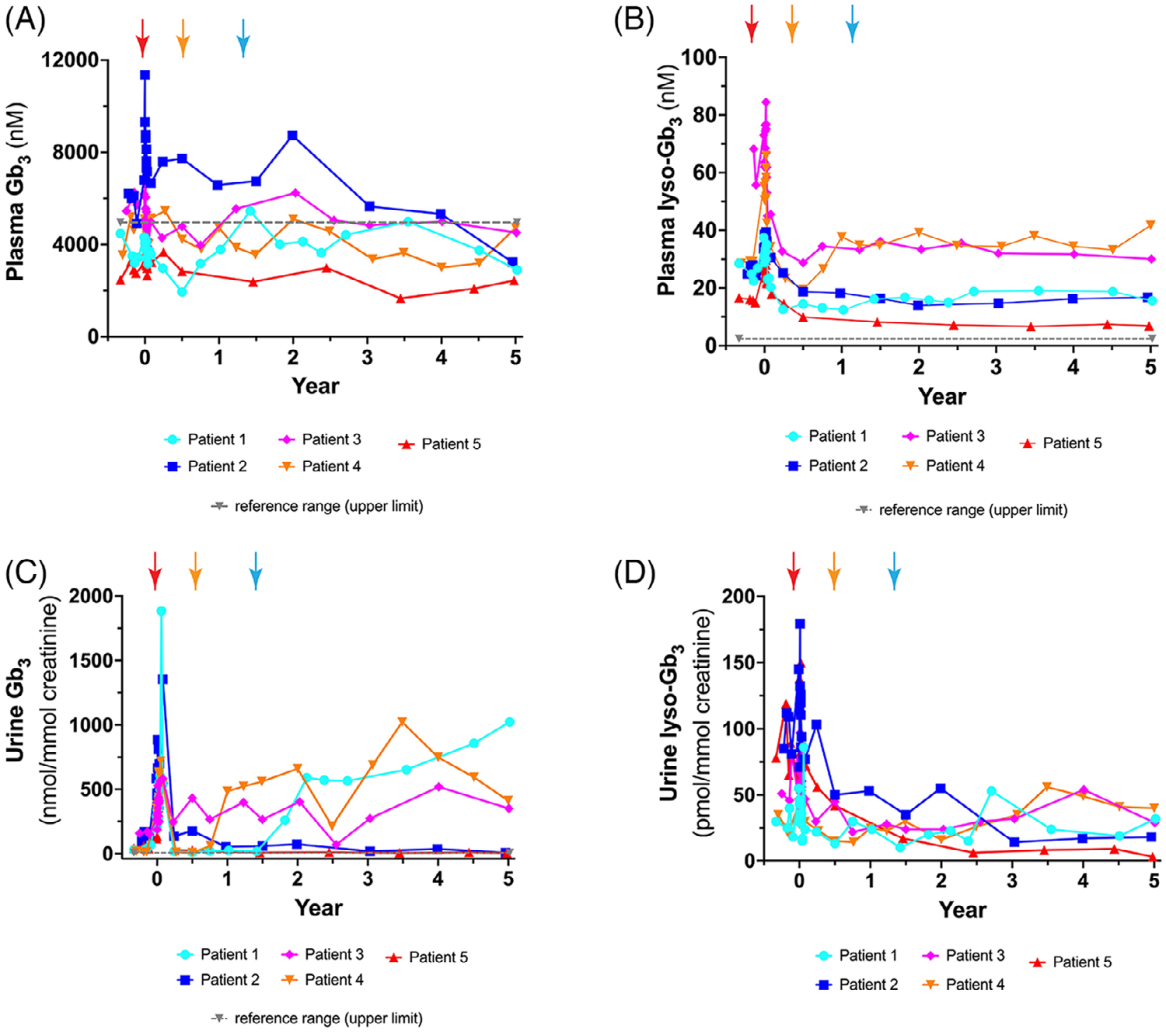
Plasma and urine globotriaosylceramide (Gb_3_) and Lyso‐Gb_3_ levels. (A) Plasma Gb_3_, (B) plasma lyso‐Gb_3_, (C) urine Gb_3_ and (D) urine lyso‐Gb_3_ levels are shown for each patient. All patients stopped enzyme replacement therapy (ET) at day −30 prior to mobilisation (red arrow). Patients 1, 2, 4 and 5 restarted ET on day 30. Patient 4 stopped ET at day 214 (year .59; orange arrow) and the Patient 1 stopped ET at day 548 (year 1.50), indicated by the blue arrow. Patient 3 elected not to restart ET after infusion of transduced cells. The upper limit of the reference range is shown for each metabolite, with the exception of urine lyso‐Gb_3_ which was not detected in a cohort of normal individuals. The upper limit of plasma Gb_3_ is 4961 nM, plasma lyso‐Gb_3_ is 2.4 nM and urine Gb_3_ is 7.2 nmol/mmol creatinine.

Plasma Gb_3_ levels decreased throughout the course of this study; all five patients had plasma Gb_3_ levels within the reference range at year 5 (Figure 4A). Note that Patient 1 stopped ET on day 548, Patient 4 stopped ET on day 214 and Patient 3 did not resume ET after transplantation of transduced cells. Interestingly, there were no significant differences in plasma Gb_3_ in any patient observed before and after the gene therapy treatment (Supporting Information Figure 8).

**FIGURE 5:**
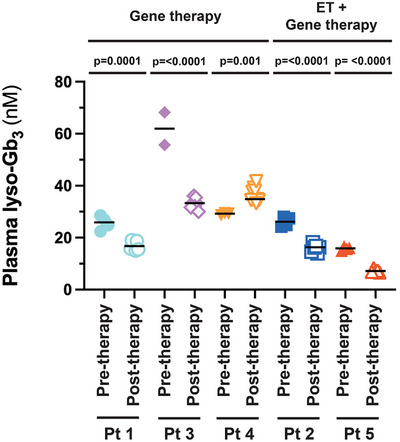
Plasma lyso‐Gb_3_ levels (nM). Dot plots are shown for plasma lyso‐Gb_3_ levels for each patient. Data points shown before pre‐treatment include samples collected during Phase 1 (screening) and Phase 2 (pre‐mobilisation). Post‐treatment samples are analysed from years 1 to 5, with the exception of Patient 1 when samples were collected once this subject stopped enzyme replacement therapy (ET) at year 1.50. Patients 1, 3 and 4 underwent an ET pause (gene therapy), while Patients 2 and 5 elected to remain on ET therapy (ET + gene therapy). Unpaired *t*‐tests were performed. The upper limit of plasma lyso‐Gb_3_ is 2.4 nM.

Plasma lyso‐Gb_3_ is a recognised metabolite of interest in Fabry disease. Elevated serum lyso‐Gb_3_ correlates with disease outcome and it is a more soluble metabolite than serum Gb_3_.^21^, ^22^ On the other hand, it is present in much lower concentrations. Plasma lyso‐Gb_3_ levels were stable from years 1 to 5 in all patients (Figure 4B). Analysis of plasma lyso‐Gb_3_ levels before and after gene therapy revealed statistically significant decreases of plasma lyso‐Gb_3_ in Patient 1 (gene therapy alone), Patient 2 (ET + gene therapy), Patient 3 (gene therapy alone) and Patient 5 (ET + gene therapy; Figure 5). Interestingly, Patient 4 showed a small, but significant increase in plasma lyso‐Gb_3_ (pre‐therapy = 29.09 nM; post‐therapy = 36.31 nM).

Urine Gb_3_ levels varied depending on whether patients selected post‐treatment ET therapy or not (Figure 4C). Urine Gb_3_ increased when each patient stopped their biweekly ET administration (Figure 4C). Patient 1 showed a biphasic urine Gb_3_ curve: a sharp increase until year 2.1, followed by a more gradual increase until year 5. Urine Gb_3_ for Patient 3 remained elevated but fairly constant throughout the follow‐up period. Patient 4 showed elevation of urine Gb_3_ until 3.5 years post‐transplantation, followed by a decrease until year 5. In contrast, Patients 2 and 5 remained on ET and their urine Gb_3_ levels remained close to baseline levels (Supporting Information Figure 9). Differences in urine Gb_3_ were observed to be statistically significant in both cohorts: both for the group that was on gene therapy alone and the two patients on ET and gene therapy (Supporting Information Figure 10).

Urine lyso‐Gb_3_ levels fluctuated throughout the year 1–5 period, but remained relatively low (Figure 4D). Interestingly, there was a significant decrease in urine lyso‐Gb_3_ in Patients 2 and 5 pre‐ and post‐therapy who were on both ET and gene therapy (Supporting Information Figure 11). The evaluation of the complete biomarker profiles (with all the isoforms and analogues) for these five Fabry patients has recently been published by our group.^12^

### 2.6 Antibody titre

**FIGURE 6:**
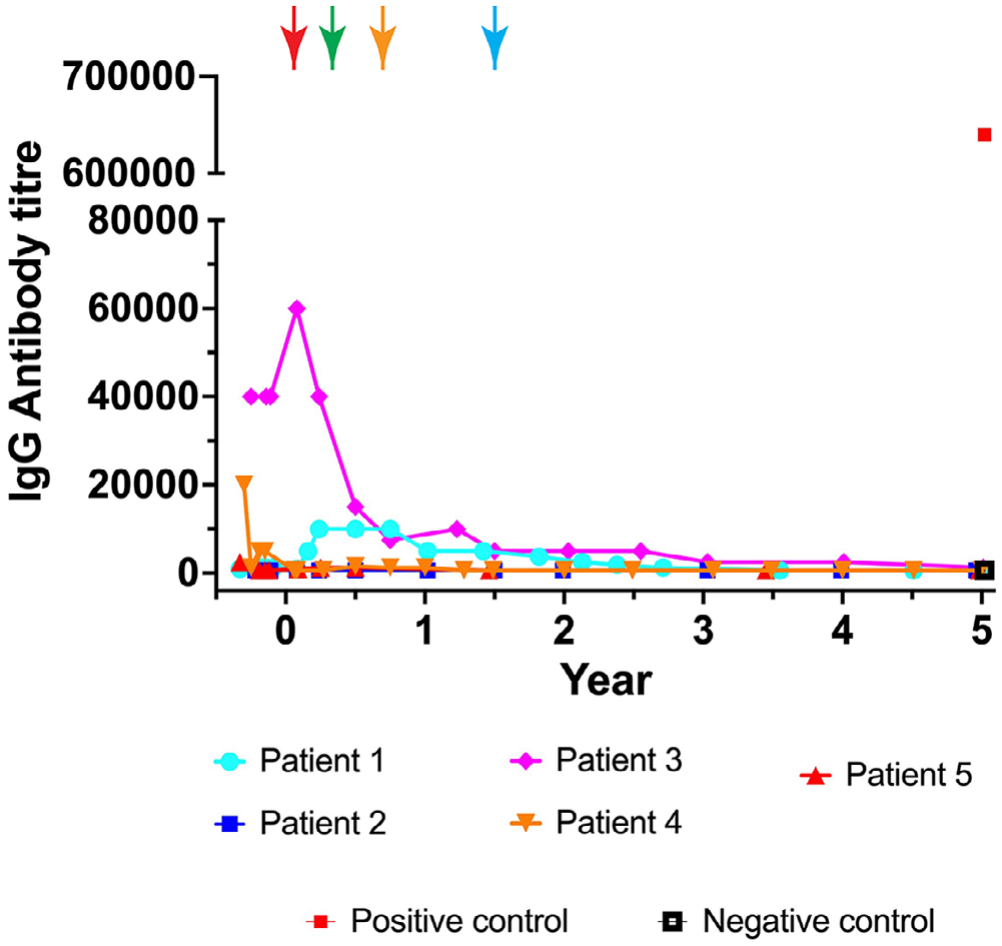
Antibody titre. Enzyme‐linked immunosorbent assays (ELISA) were completed at each time point as illustrated. The red arrow is at day −30 when enzyme replacement therapy (ET) was stopped prior to mobilisation. The green arrow is at day 30 when ET was restarted for Patients 1, 2, 4 and 5. The orange arrow depicts when Patient 4 stopped ET at day 214 (year .59) and the blue arrow is when Patient 1 stopped ET at day 548 (year 1.50). Patient 3 elected not to restart ET after stem cell transplant. The positive control sample represents serum from a patient receiving ET with a robust anti‐agalsidase response, whereas the negative control sample is serum from a patient with no immune response to the exogenous α‐galactosidase A (α‐gal A) enzyme.

Antibodies that recognise recombinant α‐gal A protein are a significant impediment to ET treatment. Patients with elevated immune‐associated reactions were excluded from this FACTs study. As we reported earlier,^11^ there was an initial transient rise in anti‐α‐gal A antibody titre in Patients 1, 3 and 4. However, this reactivity decreased in the early stages of the trial.^11^ We now illustrate that anti‐α‐gal A antibody titres remain at or near baseline beyond 18 months in this study (Figure 6).

### 2.7 Outcome correlations

We graphed outcome parameters including plasma α‐gal A, leukocyte α‐gal A, plasma Gb_3_, plasma lyso‐Gb_3_, urine Gb_3_, urine lyso‐Gb_3_ with other variables including VCN, cells/kg and age at transplant. We selected a correlation coefficient of .8–1.0 for presentation. Plasma α‐gal A correlates with leukocyte α‐gal A (Supporting Information Figure 12A), VCN (Supporting Information Figure 12B) and age at transplant (Supporting Information Figure 12C). In addition to plasma α‐gal A, leukocyte α‐gal A correlates only with VCN (Supporting Information Figure 12D). Finally, VCN correlates to age at transplant (Supporting Information Figure 12E), in addition to plasma α‐gal A and leukocyte α‐gal A, as discussed above. Notably, there was no correlation between any of the other parameters for the five patients treated in this clinical trial (data not shown).

### 2.8 Adverse events

Only two adverse events (AEs) were reported since our previous publication.^11^ Patient 2 had one incident of vomiting (Grade 2) and one report of wheezing/asthma (Grade 2; Table 1). Only two severe adverse events (SAEs) were documented during the 5‐year duration of this clinical trial: febrile neutropenia in one patient and a catheter‐related infection in a second patient. Both events occurred during treatment phase and were detailed in our initial report.^11^ Treating patients with autologous CD34+ cells engineered to express α‐gal A continues to be a safe therapy for Fabry disease patients.

**TABLE 1:** Adverse events.

Event	Number	Grade	1	2	3	4	5	Relation to study procedures^a^	Relation to protocol treatment^b^
Vomiting	1	2		X				Unrelated	Unrelated
Wheezing/asthma	1	2		X				Unrelated	Unrelated

Adverse events documented since publication of the interim report^11^ are listed. The event, number of occurrences, grade, patient number and classification are shown.

^a^ Attribution to study procedures in the context of this study refers to all study procedures such as apheresis, bone marrow transplantation procedure, blood draws, insertion of central lines and so forth as well as any protocol‐specified drugs used during the procedures such as administration of G‐CSF, melphalan and/or plerixafor.

^b^ Attribution to protocol treatment in the context of this study refers to only the gene therapy product or the autologous CD34+ cells expressing α‐galactosidase A (α‐gal A).

## 3 DISCUSSION

We have shown that LV‐mediated gene therapy employing autologous, peripheral blood‐mobilised CD34+ HSPCs with non‐myeloablative conditioning is a safe treatment modality in a Phase 1 cohort of five male Fabry disease patients. Importantly, our high‐titre vector preparation and reasonable multiplicity‐of‐infection allowed us to treat all five patients with the same good manufacturing practice‐grade batch of recombinant LV. This helped to considerably reduce costs and logistical complexity. After reduced‐intensity melphalan treatment, all patients engrafted their autologous modified α‐gal A expressing cells. Importantly, all patients synthesised and secreted α‐gal A throughout the course of the study. Expression of α‐gal A resulted in a decrease in plasma lyso‐Gb_3_ in four of five patients and stabilisation of kidney symptoms in all patients. This gene therapy approach is safe as only two SAEs were described throughout the course of this 5‐year study.^11^ Throughout the long‐term follow‐up interval of 5 years, there were no AEs of any grade attributable to the cellular gene therapy intervention or host conditioning. We demonstrate that this therapeutic approach has merit, is durable and should be explored in a larger clinical trial. While this study treated only five male Fabry patients, the fact that all five treated patients responded positively to the gene therapy bodes well for outcomes in a larger study.

All five patients synthesised and secreted α‐gal A throughout this study. Plasma α‐gal A was below the reference range in four of five patients. Similarly, three of five patients had leukocyte α‐gal A‐specific activity below the reference range. Patient 2 had leukocyte α‐gal A‐specific activity within the reference range. Plasma and leukocyte α‐gal A enzyme activity remained above the reference range in Patient 5 throughout this clinical trial. Previous model system studies showed no adverse effects of supraphysiological α‐gal A expression in transgenic mice.^23^, ^24^ The elevated expression of α‐gal A in Patient 5 will continue to be monitored in our subsequent long‐term follow‐up study, but is not now a point of clinical concern. The low, but durable level of α‐gal A synthesis and secretion observed in Patient 1 is still effective in the reduction of plasma lyso‐Gb_3_. Interestingly, this patient continued to have reduced urinary protein throughout the trial, resulting in a positive eGFR slope of .09. All five patients qualified to cease their ET therapy and three of five elected to do so.

This was a safety study and not powered to evaluate the correlation between gene therapy and any specific clinical parameters. We showed stable and prolonged expression of LV‐transduced cells with no evidence of formation of anti‐α‐gal A antibodies, beyond 18 months post‐transplant. In addition, all patients are clinically healthy. Not all patients showed the same responses in catabolites but work equating specific biomarkers and clinical outcomes in Fabry disease is still in its early stages. This is also evident from the lack of LGE advancement in any patient despite some increases in troponin levels and stabilisation of proteinuria despite a rise in urine Gb_3_ in Patient 1. While Patient 1 developed intermittent atrial fibrillation with an atrial thrombus, paroxysmal atrial fibrillation can be seen in 13.3% of males with Fabry disease with a mean age of presentation of 67 ± 11 years suggesting that prevalence increases with age.^25^, ^26^

Success of haematopoietic cell‐based gene therapy requires conditioning with a chemotherapeutic agent to allow efficient engraftment of engineered autologous cells.^27^ Since we were treating a chronic LSD, for which ET is the current standard‐of‐care, we elected to use a reduced dosage of melphalan, opting for partial ablation. We were also cognizant that metabolic cooperativity or cross‐correction in LSDs like Fabry disease may decrease the threshold for α‐gal A activity needed.^15^, ^28^ We reasoned that this reduced‐intensity conditioning approach would lead to fewer SAEs, which would be better tolerated by patients and allow the gene therapy to be administered as an out‐patient procedure. Polyclonal reconstitution was observed in all patients up to 18 months post‐infusion.^13^ This therapy was delivered in an out‐patient manner for four of five patients; one patient was kept overnight by the hospital for observation.

Busulfan is the current chemotherapeutic agent of choice for haematopoietic cell‐based gene therapy treatment of paediatric autoimmune and malignant diseases with fatal outcome.^27^, ^29^, ^30^, ^31^, ^32^, ^33^, ^34^ Delivery of busulfan must be closely monitored and use of this agent for full ablation requires intensive care unit (ICU) admittance. Busulfan is known to cause seizures in recipients^35^ and its use can lead to long‐term gonadal toxicities^36^ along with an increased risk of secondary malignancies.^37^, ^38^, ^39^ While a stronger myeloablation regimen has been used in malignant haematopoietic diseases, we hypothesised that for a metabolic disease, stronger myeloablation with an autologous transplant was unnecessary since even partial improvements in enzyme levels may be sufficient to displace the requirement of ET. Our results support the hypothesis that high‐dose chemotherapy conditioning regimens may not always be necessary in autologous lentiviral‐based gene therapy.

Plasma Gb_3_ levels decreased in all five patients on ET, from years 1 to 5. However, these differences were not statistically significant. While elevated plasma Gb_3_ is useful for diagnosis of Fabry disease, it does not seem to be informative as a biomarker with ET treatment or chaperone therapy.^40^, ^41^ Higher serum lyso‐Gb_3_ levels were shown to be independently associated with serum creatinine and cardiomyopathy in a cohort of 69 Fabry disease patients, including 28 males.^22^ In a follow‐up report, plasma lyso‐Gb_3_ was also demonstrated to be a factor associated with adverse clinical outcomes in a long‐term study of 26 male and 40 female Fabry disease patients.^21^ Plasma lyso‐Gb_3_ was significantly lower in Patients 1–3 and 5 in our FACTs trial. Post‐treatment plasma lyso‐Gb_3_ from years 1 to 5 was elevated compared to pre‐treatment values in Patient 4. Further characterisation of plasma lyso‐Gb_3_ in Patient 4 revealed that this metabolite increased 23% compared to baseline during years 1–5 of follow‐up post‐therapy, however, urinary protein in Patient 4 remained in the reference range and eGFR was indicative of Type I chronic kidney disease. This subject has daily urinary protein secretion near the upper limit of the reference range (.15 g/day; slope = .003) and an eGFR slope of −1.80. Given the proposed clinical importance of elevated plasma lyso‐Gb_3_, this patient is a candidate to resume ET in the near future.

Interestingly, we observed that urine Gb_3_ levels rose in all three patients that paused ET (Supporting Information Figure 9). The reason(s) underscoring this observation are unclear. Increases in urine Gb_3_ have been observed either as a consequence of decreased ET dosage or the emergence of anti‐α‐gal A antibodies for patients on ET therapy.^42^ No increases in anti‐agalsidase antibody activity were observed in any patient (Figure 6), suggesting that the increased urine Gb_3_ was associated with the low, but detectable α‐gal A enzyme produced by these three patients. Despite the rise in urine Gb_3_, kidney function remained normal in all three patients that paused ET with eGFR exceeding 75 in each and decreases observed in urinary protein secretion in Patients 1 and 3. In contrast, statistically significant decreases in pre‐ and post‐treatment urine Gb_3_ and urine lyso‐Gb_3_ were observed in Patients 2 and 5. Both of these patients remained on ET after the stem cell transplantation. Collectively, these data suggest that biweekly ET infusion is useful for decreasing these catabolites, however, the absolute lowest levels are achieved by the combination therapy; with the gene therapy continuously supplying α‐gal A to each patient.

We examined whether there were correlations in enzyme activity, VCN, Gb_3_ metabolites, stem cell dosage at infusion and age at transplant, with a selected cut‐off of *R* ^2^ > .80. Plasma α‐gal A, leukocyte α‐gal A and VCN were highly correlated. Plasma α‐gal A and VCN correlate to age at transplant. There were no correlations of enzyme or VCN with Gb_3_ or lyso‐Gb_3_ in plasma or urine, underscoring the challenges of using these measures as bone fide Fabry disease biomarkers.

This was the first gene therapy clinical trial to be completed for Fabry disease.^11^, ^13^ In addition to existing gene therapy protocols, there is the potential to examine other means of introducing α‐gal A into circulation and then into target cells. Our group has recently shown that Fabry patient T cells can be appropriate targets for implementing therapy, utilising our LV‐based protocol.^43^ These autologous cells can be collected easily, expanded, transduced and infused multiple times as needed in out‐patient procedures. The transduced T cells distribute the enzyme systemically throughout the body. Transduced T cells can also be long‐lived in the body and take up residence at key sites. We have shown that analogous vector‐transduced T cells may also be effective for other LSDs, including Gaucher, Farber and Pompe diseases.^43^ This concept of an alternate autologous cell source is especially timely given how adept transplant groups have become at collecting, transducing and expanding T cells for chimeric antigen receptor (CAR‐T)‐based cancer trials. Furthermore, implementation of an autologous T cell‐based gene therapy protocol to treat eligible Fabry patients may cost even less than the HSPC‐based protocol we used here.

Preclinical testing of AAV vector backbones to deliver *GLA* cDNA has been reported.^44^, ^45^ These studies have led to the development of clinical trials (NCT04046224 and NCT040400409), although the latter trial has been terminated. Two additional groups are also utilising analogous AAV vectors to introduce the *GLA* cDNA into Fabry patients (NCT04519749 and NCT06270316).

In summary, we have shown that utilising LV‐mediated gene therapy targeting HSPCs is a safe and efficacious means of treating Fabry disease. Five males with four distinct classical Fabry disease mutations were selected to participate in this clinical trial. Gene therapy was well tolerated with only two SAEs documented to date. All five patients were eligible to pause ET and three patients elected to do so. Compellingly, we estimate that pausing ET in these three patients alone has led to a savings of $4.8 million dollars to the Canadian health care system at the time of the last patient visit of Patient 5. This cost saving continues to increase daily as all five patients are now in long‐term follow‐up for 15 years. These data also demonstrate that our LV‐mediated approach can be successfully performed in an academic setting. Our multi‐site model illustrates that cells can be collected at one site, isolated and enriched at another, transduced at a third site and shipped back to the origin for infusions into patients. We have demonstrated that assays can also be performed at multiple locations. Canada has the ability to incorporate such true experimental, ‘bench‐to‐bedside’ approaches directly into the national health care system. There is already an example of such cooperative studies in Canada for individualised CAR‐T cells engineered against CD19+ cancers.^46^ Another approach would be to form an alliance of University hospitals to manufacture gene therapies, as has been done in Switzerland.^47^ Collectively, can the lessons learned from CAR‐T cell therapies for treating cancer, for example, be applied to rare diseases including LSDs such as Fabry disease? Future clinical trials in this area would aim to expand the patient cohort examined in this study as well as include female Fabry disease patients and younger male subjects.

## 4 MATERIALS AND METHODS

### 4.1 Patients

Male Fabry disease patients were recruited to this multiple‐centre, single‐arm Phase 1 clinical trial from Calgary, AB; Halifax, NS; and Toronto, ON, from September 2016 to October 2018. Subjects were screened and provided written informed consent prior to recruitment to this study. Recruitment was restricted to men (age 18–50) with confirmed Type 1 classical phenotype Fabry disease confirmed by *GLA* genotyping who had received ET for at least 6 months prior to study enrolment. Inclusion criteria comprised an eGFR, >45 mL/min/1.73 m^2^ (chronic kidney disease epidemiology collaboration equation [CKD‐EPI]) and left ventricular ejection fraction >45%. Patients with advanced Fabry disease were excluded. Additional eligibility criteria were previously reported.^11^ Ozmosis Research, Inc. (Toronto, ON) assigned a unique study identifier to each patient and all patients consented to the release of de‐identified data.

### 4.2 Lentiviral vector

The LV‐AGA vector was described previously.^15^ Large‐scale, high‐titre, clinical‐grade, recombinant LV was manufactured, purified and qualified by the Indiana University Vector Production Facility, Indianapolis, IN.

### 4.3 Study design

Three regional haematopoietic stem cell centres (Calgary, AB; Toronto, ON; and Halifax, NS) participated in Fabry patient recruitment. All steps including mobilisation, apheresis, transduction, conditioning and transplantation were described in our earlier report.^11^ Briefly, two patients received filgrastim (16 µg/kg) only while three patients were administered filgrastim (16 µg/kg) and plerixafor (240 µg/kg) for stem cell mobilisation. A back‐up graft of at least 2.5 × 10^6^ unmanipulated CD34^+^ cells/kg was harvested from each patient for use in the event of graft failure. After apheresis, the nucleated haematopoietic cells were transported to the Juravinski Cancer Centre, Hamilton, ON, for CD34^+^ cell enrichment. Selected cells were then transported to the Orsino Cell Processing Laboratory, Princess Margaret Cancer Centre, Toronto, ON, for LV transduction. The transduction protocol was described in detail.^15^ After cryopreservation and safety/sterility testing, and an approved Certificate of Analysis from Health Canada, the drug product was transported back to the stem cell centre of origin. Patients received melphalan (100 mg/m^2^) intravenously 1 day prior to autologous drug product infusion. After we observed prolonged α‐gal A activity in both leukocytes and plasma of Patient 1, the protocol was amended to allow patients to discontinue ET after an appropriate consultation/consent procedure. Patient 1 was not able to attend the follow‐up by calendar date of 5 years due to COVID restrictions and attended at 5.5 years after an amendment to include these data were filed.

### 4.4 ET pause

ET pause was considered in patients that were medically stable 6–18 months post‐transplant. This included stable production of α‐gal A enzyme levels, stability of plasma and urine Gb_3_ and lyso‐Gb_3_ metabolite levels and stable characterisation of patient health status with particular emphasis on renal, cardiac and neurologic investigations. To be a candidate to cease ET, each patient was required to synthesise α‐gal A at a level deemed acceptable by the investigator, be medically stable, and provide written consent for withdrawal of ET following a discussion with their study physician. Finally, the Clinical Trials Steering Committee unanimously agreed and provided written approval that ET could be stopped for the patient after review of all relevant data and literature and evaluation of the risks versus benefits to each patient.

### 4.5 Safety and functional efficacy assessments

The National Cancer Institute Common Terminology Criteria for Adverse Events version 4.03 was used to report AEs and SAEs. Analysis of drug product efficacy included the analysis of plasma and leukocyte α‐gal A activity^15^, ^48^, ^49^; detection of Gb_3_ and lyso‐Gb_3_ from plasma and urine by tandem mass spectrometry (MS/MS)^12^, ^50^, ^51^, ^52^; presence and persistence of LV marked cells in peripheral blood^15^; anti‐α‐gal IgG antibody levels measured by enzyme‐linked immunosorbent assay (ELISA; modified from Lee et al.)^53^; clinical outcomes; and detection of VCN.  All peripheral blood enzyme activities were measured in the trough period more than 10 days after ET administration and during the 60 days bracketing the infusion of transduced cells when ET was stopped. Plasma α‐gal A activity and leukocyte α‐gal A‐specific activity reference ranges were defined by London Health Sciences Centre Clinical Biochemical Genetics Laboratory (headed by Dr. Rupar) based on 150 specimens referred for diagnostic testing. No additional bone marrow samples were drawn beyond 1 month post‐transplant, as per protocol. Cardiac echocardiograms and magnetic resonance imaging was performed as described in ref.^54^ Reference ranges for some clinical parameters varied between study sites. The reference ranges selected for presentation were selected from the Calgary site since three of five patients were recruited there and at the end of the trial four patients were being followed at this location.

### 4.6 Study oversight

The clinical trial (NCT02800070, Health Canada—approved 26 April, 2016) was conducted in compliance with the Declaration of Helsinki and local institutional and/or university Human Experimentation Committee requirements. Research Ethics Board (REB) approval was provided by all local sites (described below). This study was designed by the FACTs team. Ozmosis Research, Inc., of Toronto, Canada performed clinical trial management. Clinical data were collected by the investigators of the FACTs team who were responsible for clinical assessments during Phases 3 (treatment phase) and 4 (long‐term follow‐up). Clinical and laboratory data were reviewed monthly by the FACTs team. Clinical decisions and safety monitoring for each patient were completed by a Clinical Trial Steering Committee (CTSC) of the FACTs team. A Data Monitoring Safety Committee (DSMC) reviewed clinical and safety data after the treatment phase for Patients 1 and 3.

## AUTHOR CONTRIBUTIONS

α‐Gal A enzymatic assays were performed by the London Health Sciences Centre Clinical Biochemical Genetics Laboratory (Director C. Anthony Rupar), London, ON. Gb_3_ and lyso‐Gb_3_ analyses were performed by the Auray‐Blais laboratories at the Université de Sherbrooke, Sherbrooke, QC. Vector copy number assays and ELISAs to measure anti‐α‐gal IgG antibodies were performed by the Medin laboratories at the Medical College of Wisconsin, Milwaukee, WI. The FACTs team had confidential access to the data through the trial management company. Aneal Khan, Dwayne L. Barber and Jeffrey A. Medin wrote and edited the manuscript. All the authors edited and approved the final manuscript before publication.

## CONFLICT OF INTEREST STATEMENT

Aneal Khan holds research grants from Actelion, Amicus, AVROBIO, Canadian Institutes of Health Research, Canadian Fabry Disease Initiative, Genome Canada/Genome Prairie, Mallinckrodt, Moderna, New Frontiers in Research Fund (Canada), Resverlogix, Sangamo, Sanofi/Genzyme, Shire HGT and Takeda. He has received consulting fees, speaker fees and travel grants from Actelion, Amicus, AVROBIO, Sanofi/Genzyme and Shire HGT. Dr. Khan is the President and Medical Director of M.A.G.I.C. Clinic (Metabolics and Genetics in Canada), Chief Executive Officer of Discovery DNA and Medical Director of Maternal Genomics. He is the President of rDNA (Rare Disease Network of Alberta). Dr. Khan is part of a revenue sharing agreement with University Health Network for Fabry gene therapy.

Dwayne L. Barber and Alexandra Berger were partially paid from a Sponsored Research Agreement by AVROBIO, Inc.

C. Anthony Rupar has the following financial relationships to disclose: the Biochemical Genetics clinical diagnostic laboratory at his home institution was contracted by AVROBIO, Inc. to assay enzymes on a fee for service basis. He is the laboratory director but received no personal compensation.

Christiane Auray‐Blais has received a service contract and honoraria for biomarker analysis with AVROBIO, Inc. and a grant from CIHR.

Armand Keating has received consultancy fees from AVROBIO, Inc. unrelated to this study.

Michael L. West has received research grants, consulting fees or speaker fees with Amicus Therapeutics, Chiesi, Protalix, Sanofi and Takeda, revenue distribution agreement with University Health Network regarding gene therapy using technology from this work, shares IP in Fabry cardiac biomarker panel with University of Alberta.

Jeffrey A. Medin has the following financial relationships to disclose: Honoraria—Sanofi Genzyme and Shire; Scientific co‐Founder—AVROBIO, Inc.; Shareholder—AVROBIO, Inc.; and Grants from Canadian Institutes of Health Research and Kidney Foundation of Canada and AVROBIO, Inc.

William M. McKillop, Graeme Fraser, Daniel H. Fowler and Ronan Foley have no financial relationships to disclose in relation to this trial.

## ETHICS STATEMENT

Research Ethics Board (REB) approval was obtained from University Health Network, Alberta Health Services, Capital Health Services, Hamilton Integrated Research Ethics and the Medical College of Wisconsin Institutional Review Board for this study.

## Supporting information



Supporting Information

## Data Availability

The data that support the findings of this study are available from the corresponding author upon reasonable request.
