# Supplementary material for: Lentivirus‐mediated gene therapy for Fabry disease: 5‐year End‐of‐Study results from the Canadian FACTs trial
Source: Clin Transl Med. 2025 Jan 10;15(1):e70073. doi: 10.1002/ctm2.70073 (PMC11726700; doi:10.1002/ctm2.70073)
Supplement: Supplementary file 1 — Supporting Information [file CTM2-15-e70073-s001.pdf]

SUPPLEMENTARY FIGURE LEGENDS

Supplementary Figure 1. Plasma α-Gal A Statistical Analyses

Dot plots are shown for plasma α-gal A activity for each treated patient (Pt). Data points shown before pre-treatment include samples collected during Phase 1 (Screening) and Phase 2 (Pre-mobilization and mobilization) up to Day 0. Post-treatment samples are analyzed from years 1-5. Unpaired t-tests were performed. The mean is indicated by a black line and p-values are illustrated. The lower reference range is 5.1 nmol/min/mL and the upper reference range is 9.2 nmol/min/mL.

Supplementary Figure 2. Leukocyte α-Gal A Statistical Analyses

Dot plots are shown for leukocyte α-gal A specific activity for each treated patient (Pt). Data points shown before pre-treatment include samples collected during Phase 1 (Screening) and Phase 2 (Pre-mobilization and mobilization) up to Day 0. Post-treatment samples are analyzed from years 1-5. Unpaired t-tests were performed. The mean is indicated by a black line and p-values are indicated. The lower reference range is 24 nmol/hr/mg protein and the upper reference range is 56 nmol/hr/mg protein.

Supplementary Figure 3. Vector Copy Number (VCN) Statistical Analyses

Dot plots are shown for VCN for each patient (Pt). Data points shown before pre-treatment include samples collected during Phase 1 (Screening) and Phase 2 (Pre-mobilization and mobilization) up to Day 0. Post-treatment samples are analyzed from years 1-5. Unpaired t-tests were performed. The mean is indicated by a black line and p-values are shown.

Supplementary Figure 4. Patient Weights

Patient weight (kg) is illustrated for each treated patient during the duration of the clinical trial.

Supplementary Figure 5. Blood Pressures

Systolic and diastolic blood pressures are shown for Phases 1 and 4 for each patient: (A) Patient 1, (B) Patient 2, (C) Patient 3, (D) Patient 4, and (E) Patient 5. As Granulocyte Colony Stimulating Factor (G-CSF) and melphalan can cause changes in blood pressure during conditioning, stem cell infusion, and the neutropenic phase of BMT, these data were omitted. Linear regression was performed (black dotted line) and calculated slopes are presented.

Supplementary Figure 6. Urinary Protein Levels

Twenty-four hour urinary protein levels are shown for each treated patient: (A) Patient 1, (B) Patient 2, (C) Patient 3, (D) Patient 4, and (E) Patient 5. Lower (0 g/24 hr) and upper (0.15 g/24 hr) reference values are presented as dotted grey lines. Linear regression was performed (back dotted line) and calculated slopes are illustrated.

Supplementary Figure 7. Troponin Levels

Troponin levels are shown for each treated patient: (A) Patient 1, (B) Patient 2, (C) Patient 3, (D) Patient 4, and (E) Patient 5. Lower (0 ng/L) and upper (14 ng/L) are presented as dotted grey lines. Linear regression was performed (back dotted line) and calculated slopes are illustrated.

Supplementary Figure 8. Plasma Gb_3 Statistical Analyses

Dot plots are presented for plasma Gb_3 for each treated patient (Pt). Data points shown before pre-treatment include samples collected during Phase 1 (Screening) and Phase 2 (Pre-mobilization). Post-treatment samples are analyzed from years 1-5, with the exception of Patient 1 when samples were collected once this subject stopped ET at year 1.50. Patients 1, 3, and 4 underwent an ET pause (gene therapy), while Patients 2 and 5 elected to remain on ET therapy (ET + gene therapy). Unpaired t-tests were performed. The mean values are indicated by a black line and p-values are illustrated. The upper limit of plasma Gb_3 is 4961 nM analyzed from a pool of normal individuals.

Supplementary Figure 9. Urine Gb3 Levels in Treated Patients Undergoing an ET Pause Versus those Remaining on ET

(A) Data is presented for Patients 2 and 5 who remained on ET throughout the duration of the clinical trial. (B) Patients 1, 3, and 4 consented to an ET pause. Data is presented for these patients. Patient 1 stopped ET at year 1.50 (Day 548) (blue arrow), Patient 4 stopped ET at year 0.59 (Day 214) (orange arrow), whereas Patient 3 elected not to start ET after treatment year -0.08 (Day -30) (red arrow). The upper limit of urine Gb_3 is 7.2 nmol/mmol creatinine detected from a co-hort of normal individuals.

Supplementary Figure 10. Urine Gb_3 Statistical Analyses

Dot plots are illustrated for urine Gb_3 levels for each patient (Pt). Data points shown before pre-treatment include samples collected during Phase 1 (Screening) and Phase 2 (Pre-mobilization). Post-treatment samples are analyzed from years 1-5, with the exception of Patient 1 when samples were collected once this subject stopped ET at year 1.50. Patients 1, 3, and 4 underwent an ET pause (gene therapy), while Patients 2 and 5 elected to remain on ET therapy (ET + gene therapy). Unpaired t-tests were performed. The mean values are indicated by a black line and p-values are illustrated. The upper limit of urine Gb_3 is 7.2 nmol/mmol creatinine detected from a pool of normal individuals.

Supplementary Figure 11. Urine Lyso-Gb_3 Statistical Analyses

Dot plots are presented for urine lyso-Gb_3 levels for each patient (Pt). Data points shown before pre-treatment include samples collected during Phase 1 (Screening) and Phase 2 (Pre-mobilization). Post-treatment samples are analyzed from years 1-5, with the exception of Patient 1 when samples were collected once this subject stopped ET at year 1.50. Patients 1, 3, and 4 underwent an ET pause (gene therapy), while Patients 2 and 5 elected to remain on ET therapy (ET + gene therapy). Unpaired t-tests were performed. The mean values are indicated by a black line and p-values are illustrated.

Supplementary Figure 12. Parameter Correlation.
Plasma α-gal A activity is plotted as a function of (A) leukocyte α-gal A activity, (B) VCN, (C) age at transplant; (D) leukocyte α-gal A specific activity is graphed as a function of VCN; (E) VCN is plotted as a function of age at transplant. Patients receiving G-CSF at mobilization are shown in clear symbols. Patients who received G-CSF and plerixafor at mobilization are illustrated with solid symbols. The correlation co-efficient (R^2) is shown for each figure.

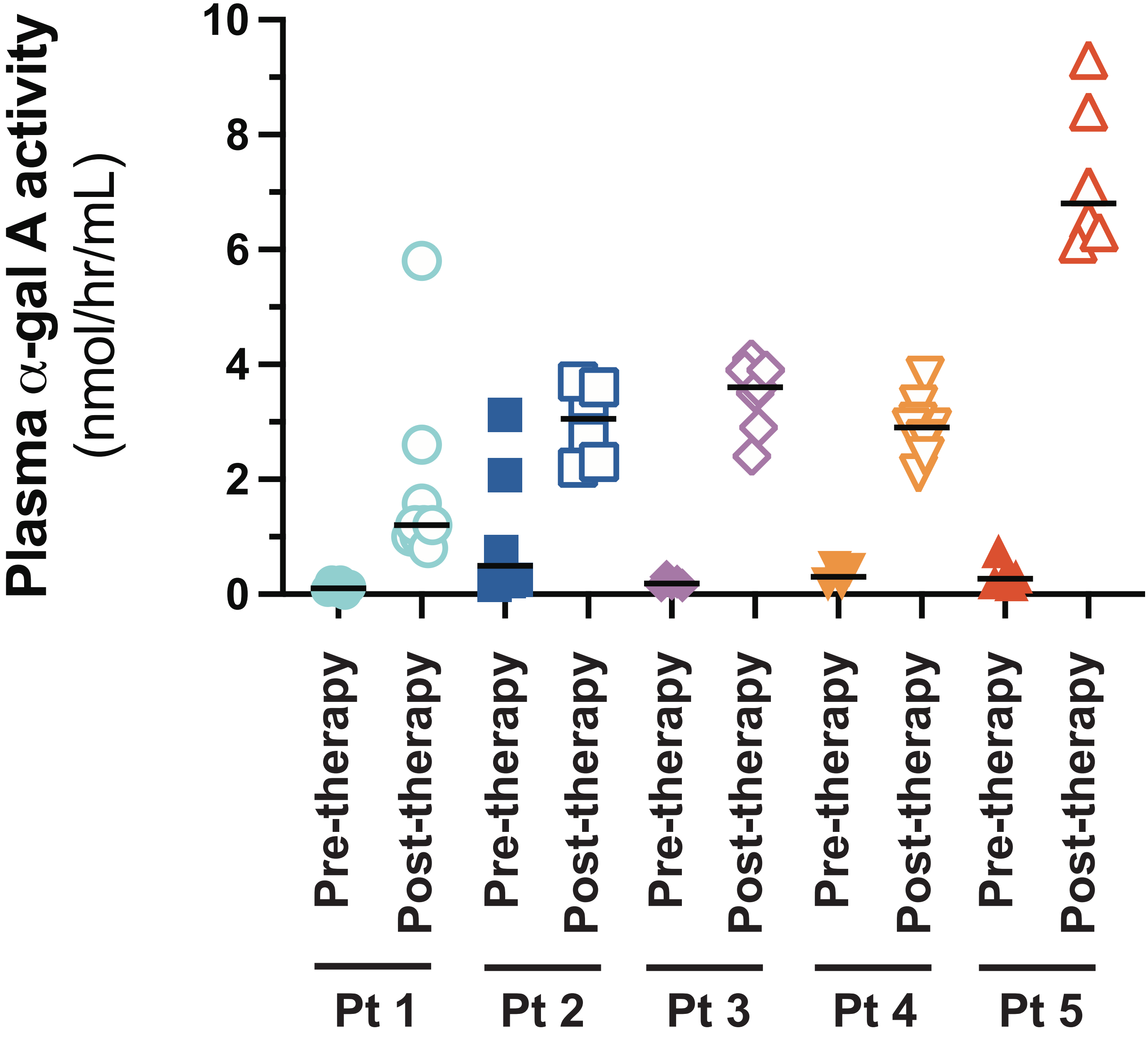

Supplementary Figure 1

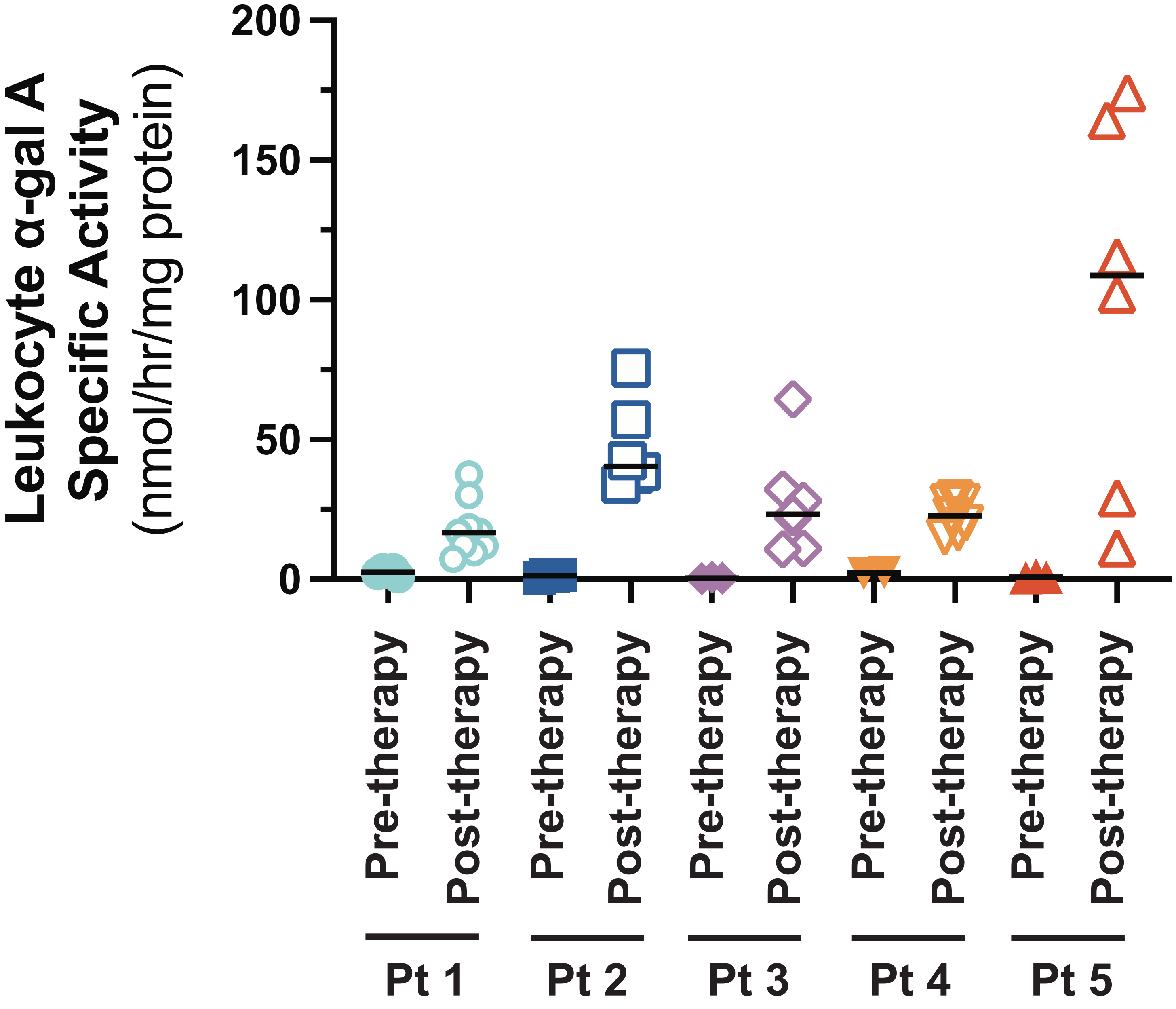

Supplementary Figure 2

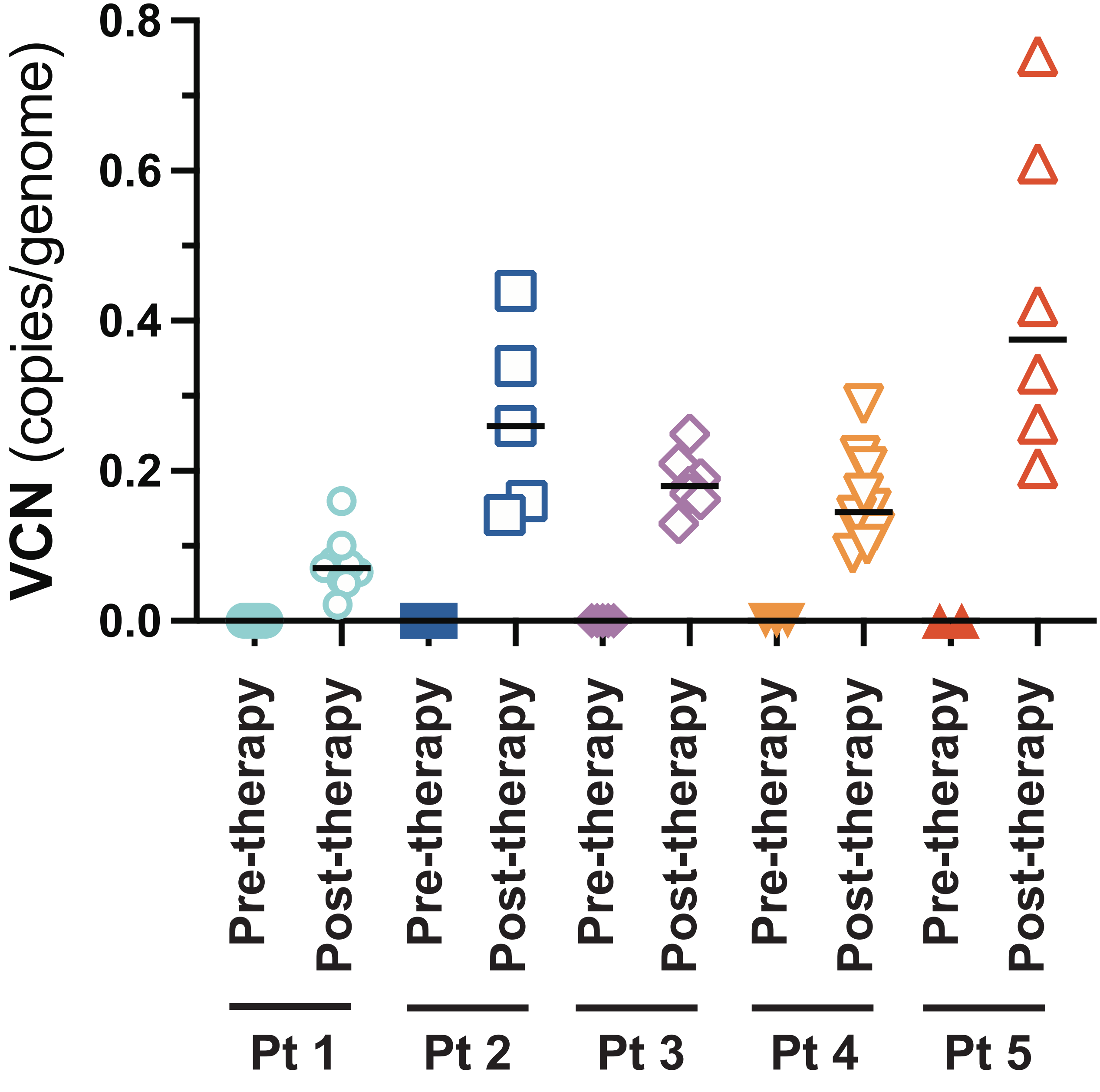

Supplementary Figure 3

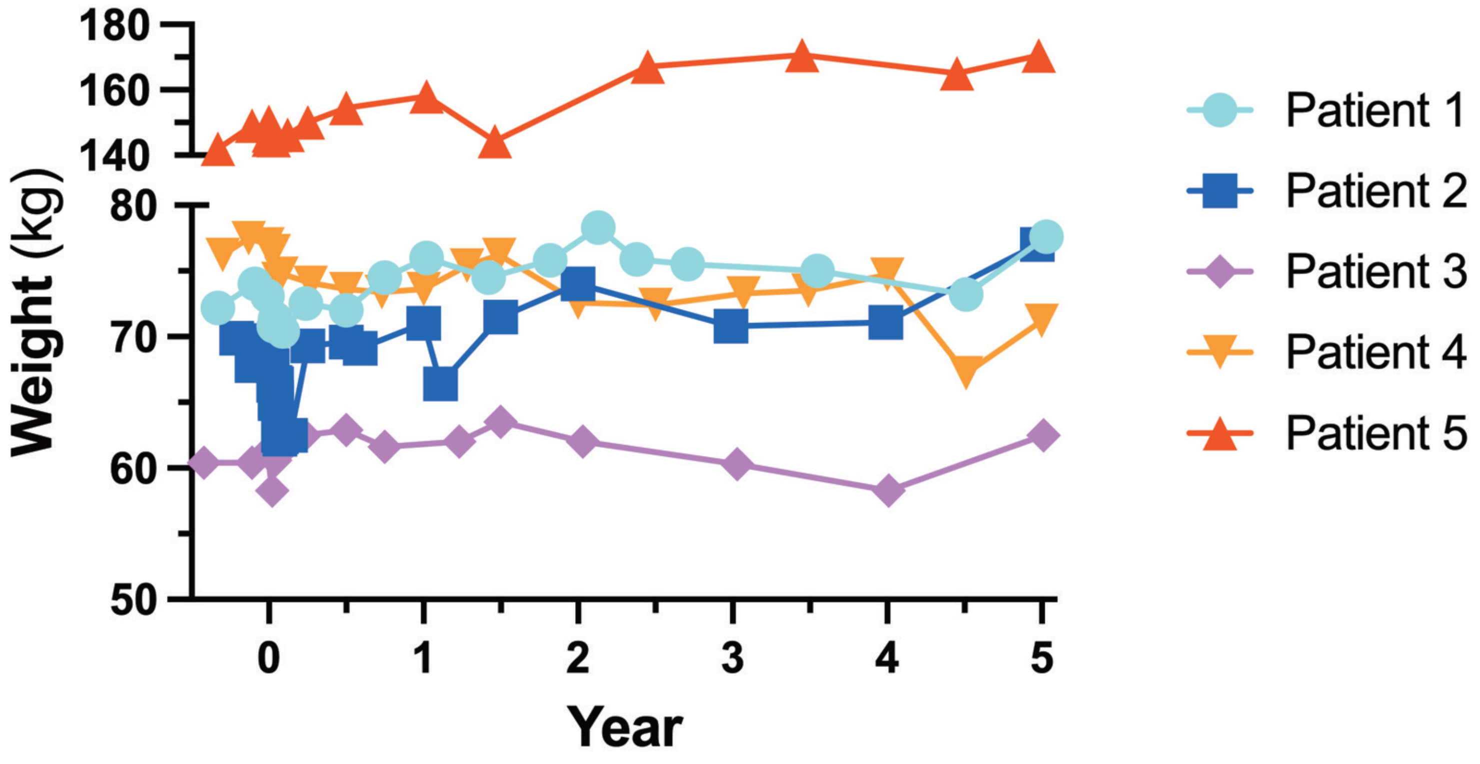

Supplementary Figure 4

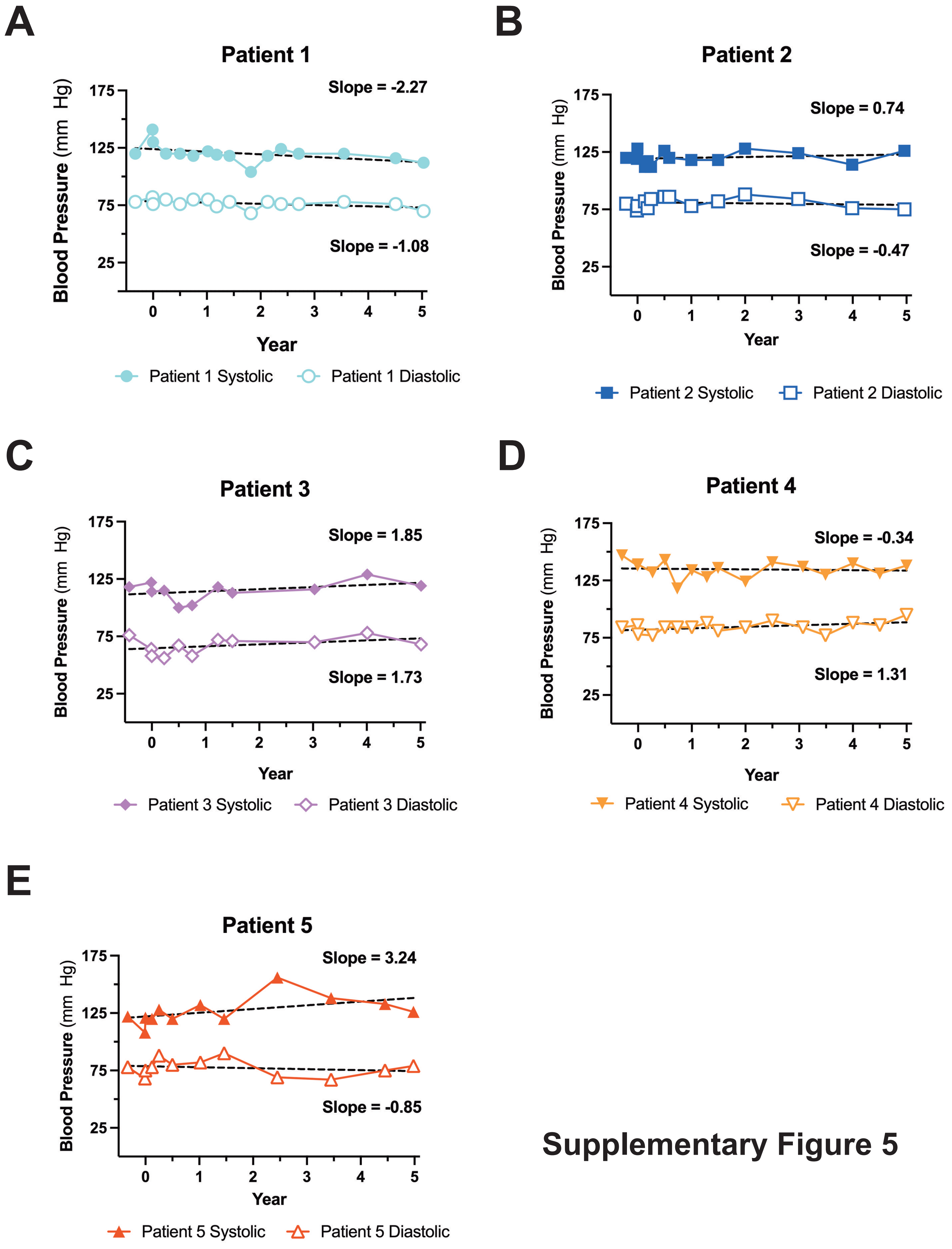

Supplementary Figure 5

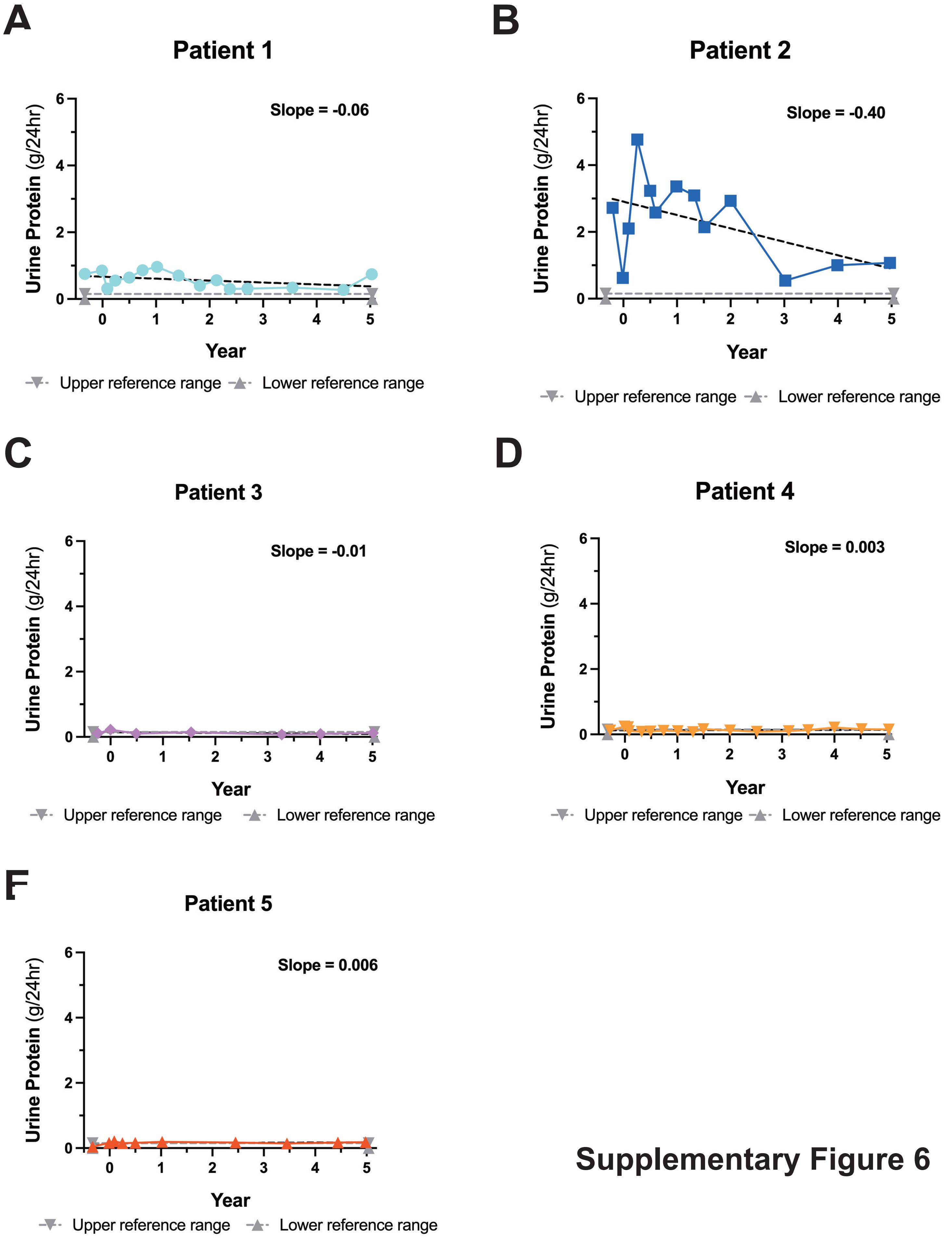

Supplementary Figure 6

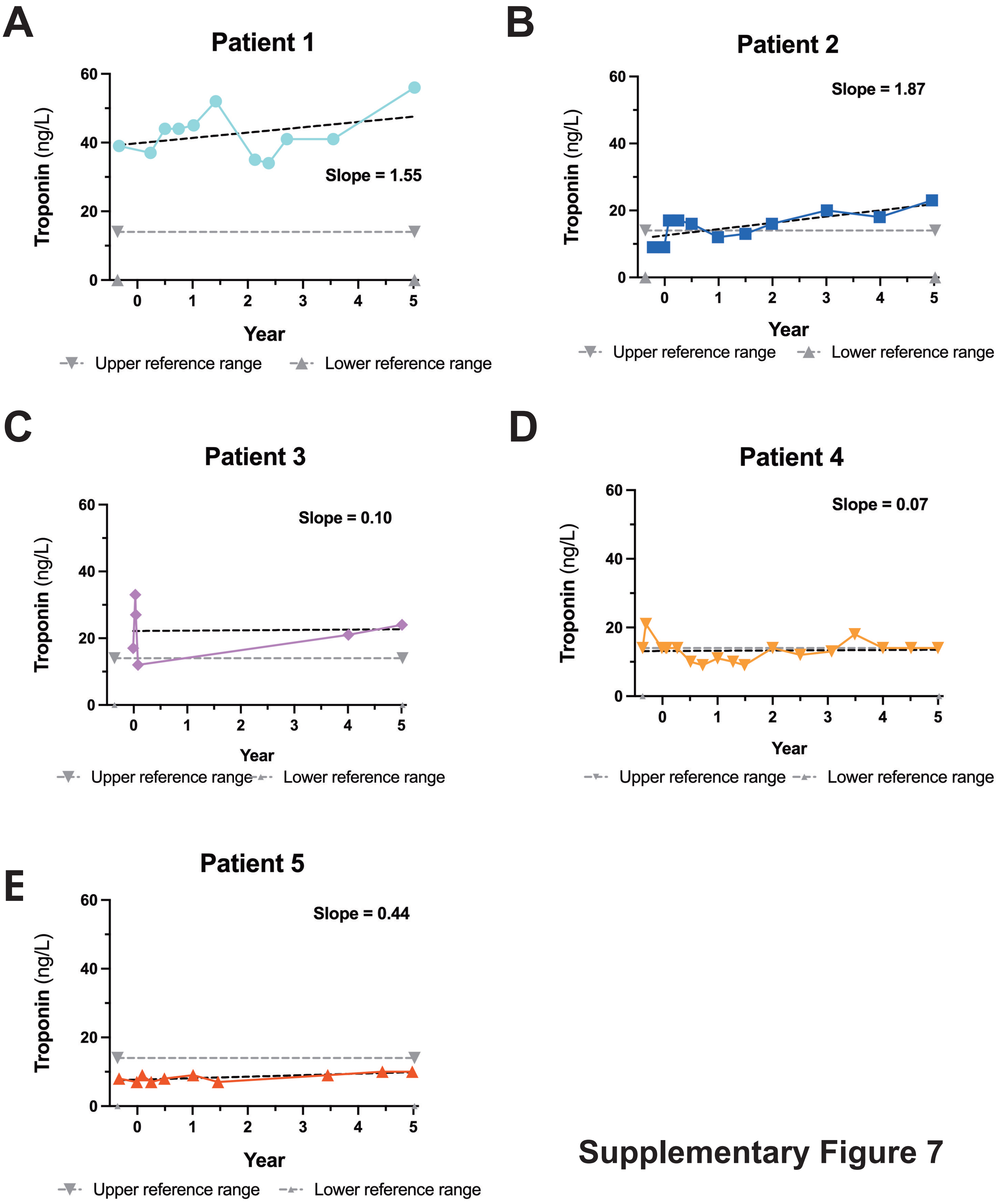

Supplementary Figure 7

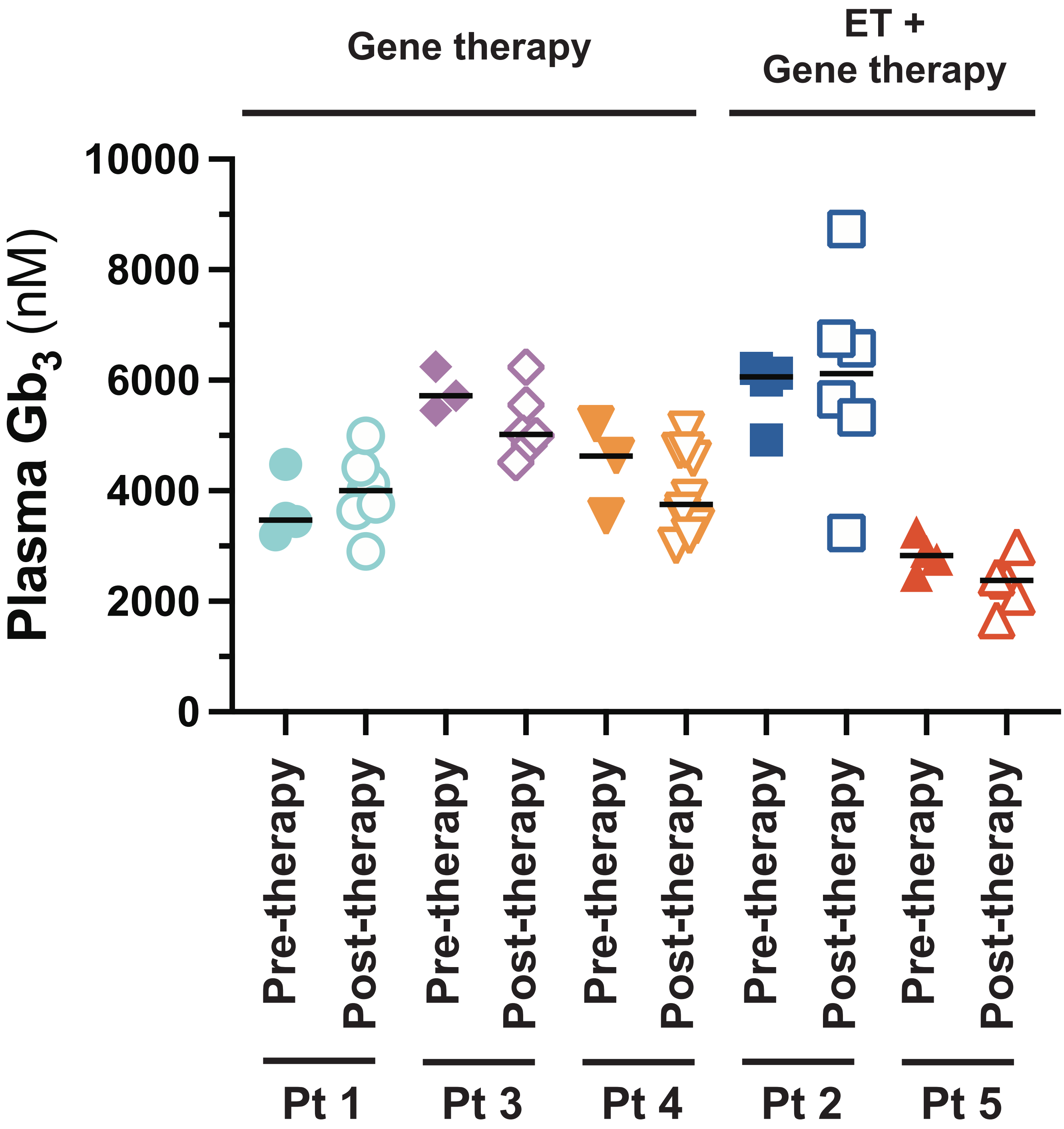

Supplementary Figure 8

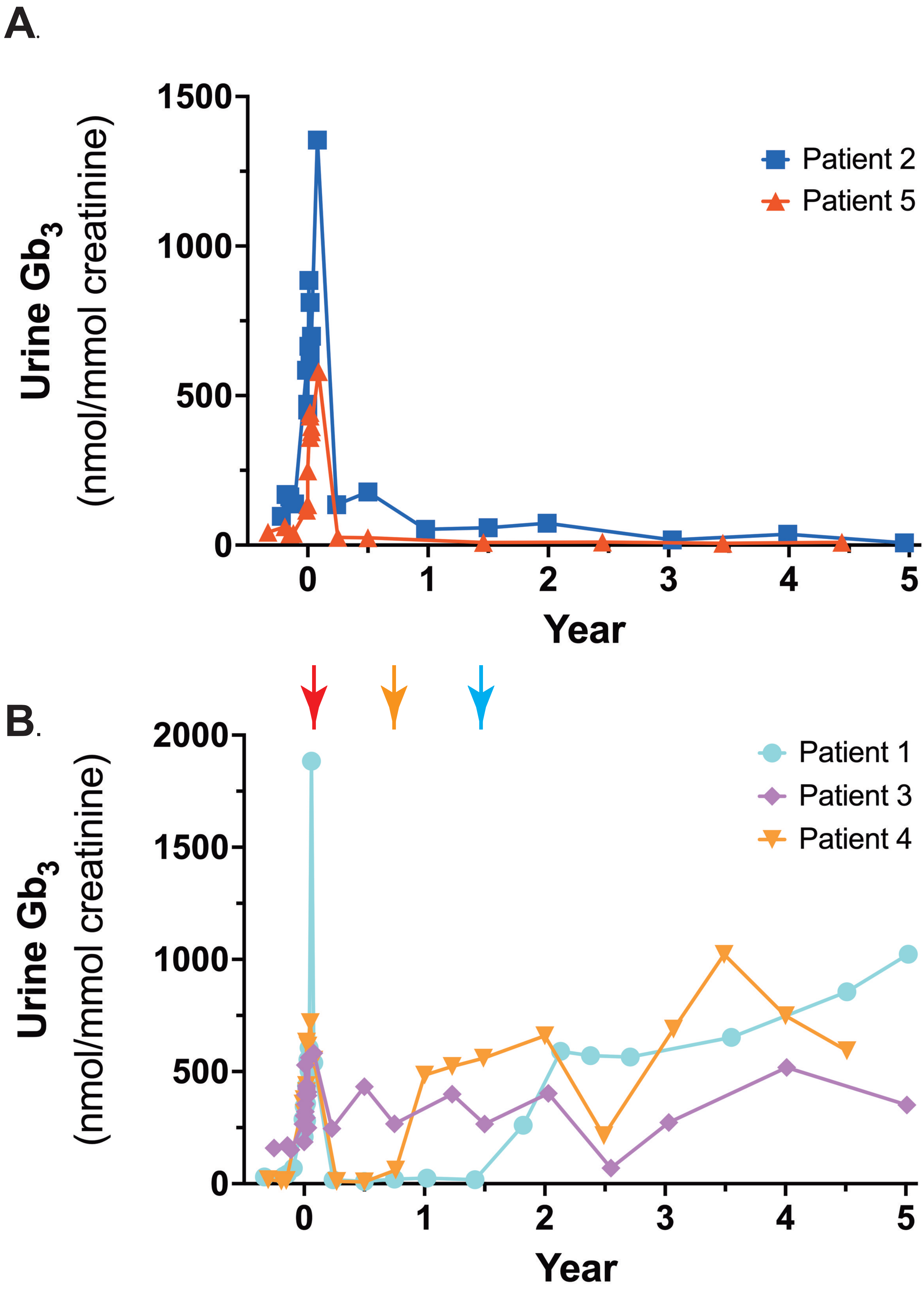

Supplementary Figure 9

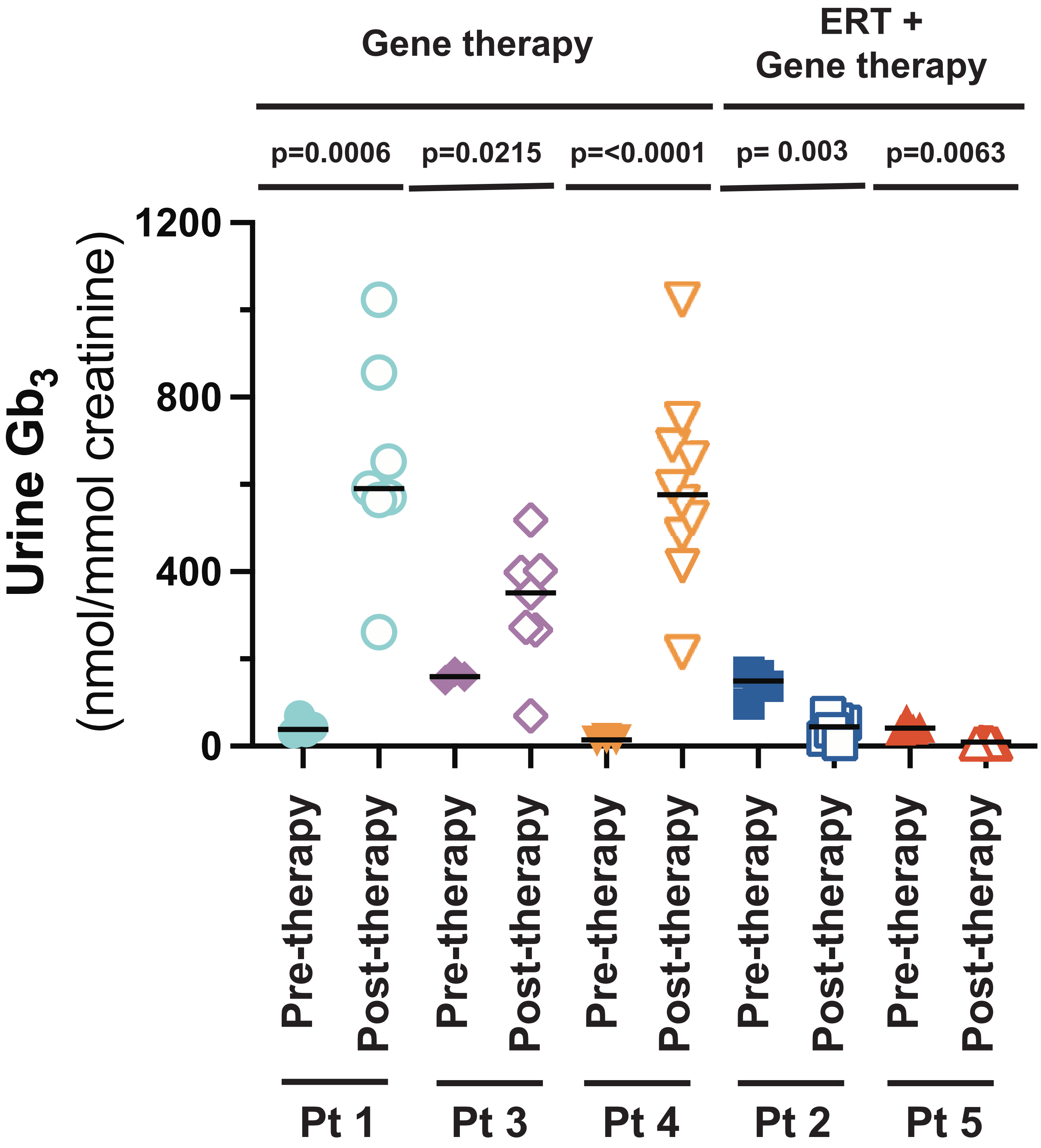

Supplementary Figure 10

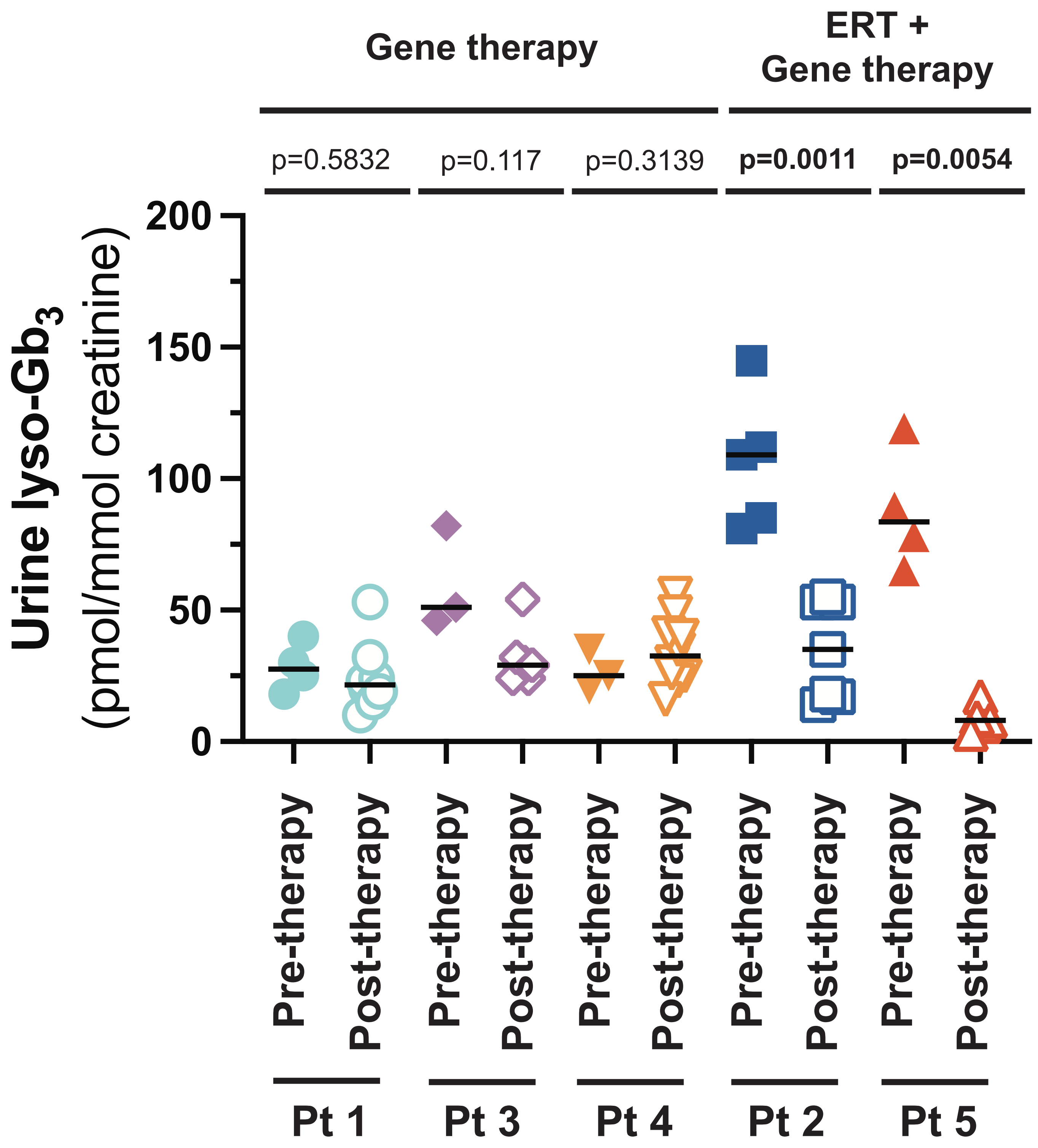

Supplementary Figure 11

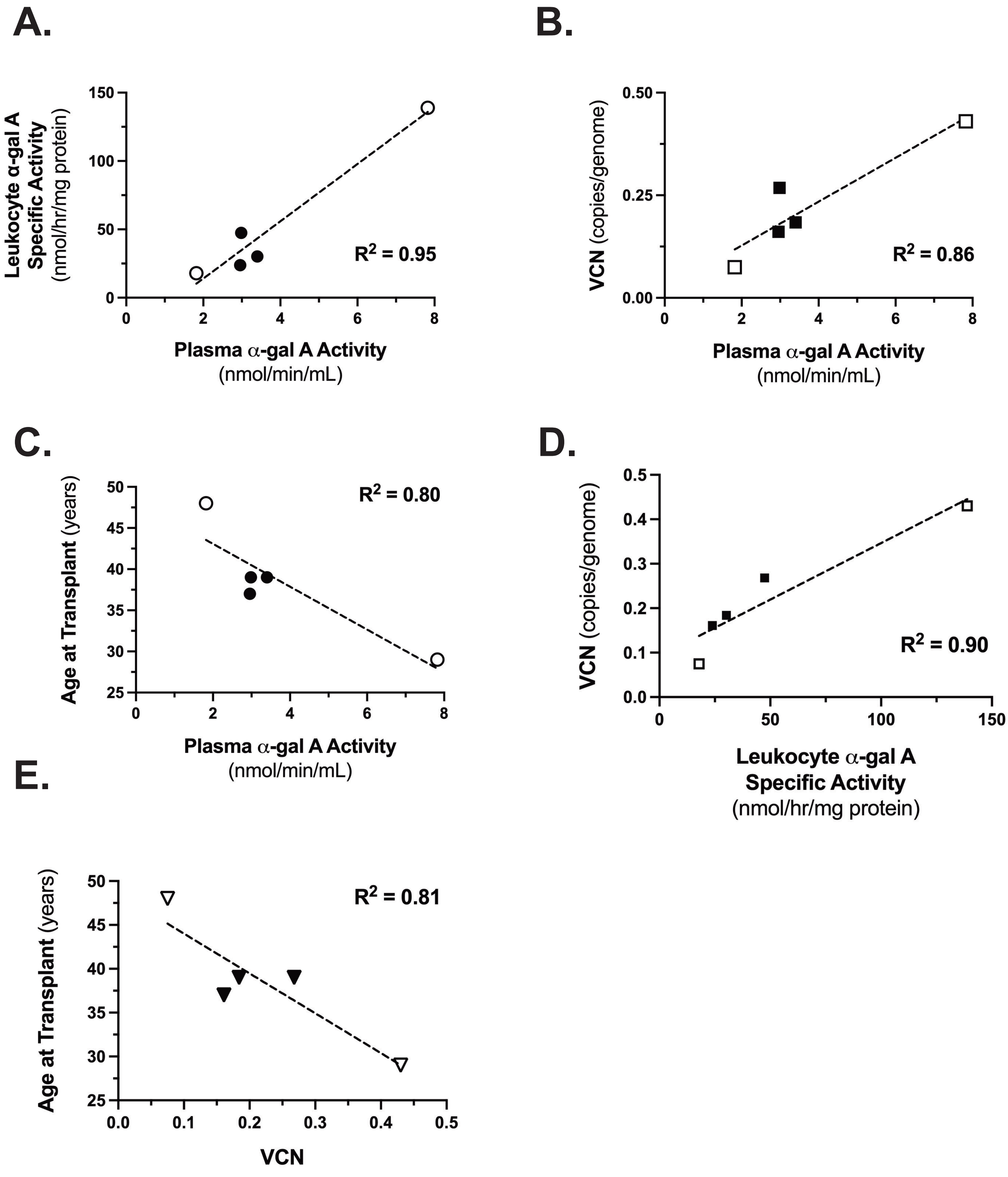

Supplementary Figure 12
